# Tuoli Tounong decoction promotes lesion resolution in granulomatous lobular mastitis by remodeling a Caspase-8-associated PANoptosis-like cell death network

**DOI:** 10.3389/fimmu.2026.1930689

**Published:** 2026-08-28

**Authors:** Yingying Yuan, Ximeng Zuo, Xiang Gao, Yue Wang, Xiaofa Li, Qian Li, Zhenzhou Chen, Xiaoguang Shi

**Affiliations:** Dongzhimen Hospital, Beijing University of Chinese Medicine, Beijing, China

**Keywords:** apoptosis, Caspase-8, granulomatous lobular mastitis, macrophage, necroptosis, PANoptosis, pyroptosis, Tuoli Tounong decoction

## Abstract

Granulomatous lobular mastitis (GLM) is a chronic inflammatory breast disease characterized by granuloma formation, abscesses, fat necrosis, and recurrent tissue injury. This study investigated whether Tuoli Tounong decoction (TTN) promotes lesion resolution in a rat model of GLM by regulating a Caspase-8-associated PANoptosis-like cell death network. Forty-two female Sprague-Dawley rats were used; six served as healthy controls, and the remaining rats underwent GLM model induction and were assigned to the model, supporting method (TF), draining method (TO), TTN, methylprednisolone (MP), or Caspase-8 inhibitor (C8i) groups. Treatments were administered for 21 days. Mammary lesions were evaluated using gross observation, histopathological staining, TUNEL staining, multiplex immunofluorescence, transmission electron microscopy, qPCR, Western blotting, flow cytometry, and ELISA. The model group showed mammary enlargement, inflammatory infiltration, granuloma formation, fat necrosis, collagen deposition, increased pyroptosis-related signaling, and macrophage/myeloid cell remodeling. TTN most effectively reduced lesion size, promoted pus drainage, preserved lobular and ductal architecture, and attenuated fat necrosis and fibrosis. Mechanistically, TTN increased Casp8 mRNA, cleaved Caspase-8, cleaved Caspase-3, and p-MLKL expression while reducing GSDMD membrane-associated accumulation and cleaved GSDMD expression. Casp8 C362S-associated amplification signals were relatively low in the TTN group. Flow cytometry suggested that TTN regulated local myeloid immune remodeling rather than broadly suppressing immune infiltration, whereas serum IL-1β and IL-18 levels showed no significant between-group differences. These findings suggest that TTN alleviates GLM lesions in association with remodeling of a Caspase-8-associated PANoptosis-like cell death network, accompanied by reduced pyroptosis-related injury and increased apoptosis- and p-MLKL-associated signals.

## 1 Introduction

Granulomatous lobular mastitis (GLM) is an uncommon chronic inflammatory breast disease that primarily involves mammary lobules. Its typical pathological features include non-caseating granuloma formation, inflammatory cell infiltration, microabscesses, fat necrosis, and recurrent local tissue destruction (1, 2). Clinically, GLM often presents as breast erythema, swelling, pain, abscess formation, ulceration, and sinus tract formation, with a prolonged disease course and a tendency to recur. Current therapeutic strategies include observation, glucocorticoids, antibiotics, incision and drainage, surgery, and traditional Chinese medicine; however, a standardized treatment strategy remains lacking, and some patients still experience prolonged treatment, recurrent lesions, extensive tissue damage, and impaired breast appearance (3–5). Therefore, it is important to clarify the mechanisms underlying persistent local inflammation, lesion chronicity, and impaired tissue repair in GLM, and to explore therapeutic strategies that promote both lesion clearance and tissue protection.

Programmed cell death plays an important role in inflammatory diseases. Previous studies have shown Caspase-1/GSDMD activation and pyroptosis-like ultrastructural changes in GLM lesions, suggesting that pyroptosis may contribute to inflammatory amplification and tissue injury in GLM (6). However, GLM lesions are characterized by complex pathological features, including inflammatory infiltration, tissue destruction, fat necrosis, and fibrotic remodeling. A single pyroptosis pathway may therefore be insufficient to fully explain lesion chronicity and resolution. PANoptosis is a recently proposed form of integrated programmed cell death, emphasizing that apoptosis, pyroptosis, and necroptosis can be cross-regulated through shared molecules and signaling complexes within the same pathological process, jointly influencing inflammatory amplification, cell clearance, and tissue repair (7). Pyroptosis is mainly characterized by Caspase-1 activation, GSDMD cleavage, and membrane pore formation, which amplify local inflammation (8). Apoptosis is a relatively low-inflammatory and regulated form of cell death that facilitates clearance of damaged cells (9). Necroptosis is mainly mediated by the RIPK1/RIPK3/MLKL pathway and has context-dependent biological effects, contributing either to inflammation or to lesion clearance and tissue repair (10). Caspase-8 has been proposed as an important regulatory node within a PANoptosis-like cell death network, and changes in its expression, activation status, and catalytic activity may collectively determine cell death fate (11). Therefore, analyzing the balance among apoptosis, pyroptosis, and necroptosis in GLM lesions from the perspective of Caspase-8-associated PANoptosis-like signaling may provide new mechanistic insights into inflammatory persistence and lesion resolution in GLM.

Recent clinical and review studies suggest that traditional Chinese medicine has potential value in the comprehensive treatment, long-term outcomes, and signaling pathway regulation of GLM (12–14). TTN is composed of multiple herbs, and its compatibility can be interpreted pharmacologically as an integrated intervention targeting immune regulation, inflammatory control, lesion clearance, and tissue repair (12–15). Astragali Radix-Angelicae Sinensis Radix is an important herb pair in this formula and is closely associated with immune regulation and tissue repair. Modern pharmacological studies have shown that Astragali Radix and astragalus polysaccharides exert immunomodulatory, anti-inflammatory, antioxidant, and antifibrotic effects and can affect immune cell proliferation, cytokine release, and macrophage functional status; Angelicae Sinensis Radix and its polysaccharides also exhibit anti-inflammatory, antioxidant, immunomodulatory, and tissue-protective activities (16, 17). Angelicae Dahuricae Radix-Gleditsiae Spina is more closely associated with inflammatory control, lesion clearance, and wound repair. Coumarins from Angelicae Dahuricae Radix inhibit the NF-κB pathway and reduce LPS-induced release of NO, IL-1β, IL-6, and TNF-alpha in macrophages; its extracts have also been shown to promote inflammatory resolution, granulation tissue formation, and angiogenesis in infected wound models (18, 19). Studies of Gleditsiae Spina have shown anti-inflammatory activity, reducing TNF-alpha, IL-6, IL-17, and IL-1β levels and improving histopathological injury (20). These pharmacological findings suggest that TTN may not function as a simple anti-inflammatory formula, but rather as a multi-herb, multitarget intervention that participates in local inflammatory regulation, lesion clearance, and tissue repair.

Although previous studies have suggested that GLM is associated with pyroptosis and that traditional Chinese medicine may modulate the local immune microenvironment and inflammatory pathways in GLM, whether TTN affects the balance among apoptosis, pyroptosis, and necroptosis through a Caspase-8-associated PANoptosis-like network remains unclear (6, 14, 15). Accordingly, this study established an SD rat model of GLM and compared the effects of TF, TO, TTN, MP, and C8i on mammary tissue injury, local immune infiltration, and programmed cell death modes. We focused on whether TTN regulates Caspase-8-associated molecular alterations to reshape the PANoptosis-like cell death network, thereby modulating the balance among pyroptosis-, apoptosis-, and necroptosis-related cell death signals and ultimately contributing to lesion resolution and mammary tissue remodeling.

## 2 Materials and methods

### 2.1 Animals and ethical statement

Forty-two healthy multiparous female Sprague-Dawley (SD) rats weighing 350 ± 10 g were purchased from Beijing Vital River Laboratory Animal Technology Co., Ltd. Rats were housed under standard barrier conditions at 22–25 °C, 50%–60% relative humidity, and a 12 h/12 h light/dark cycle with free access to food and water. Experiments were initiated after 3 days of acclimation. All animal experiments were approved by the Animal Ethics Committee of Dongzhimen Hospital, Beijing University of Chinese Medicine (approval No. DZMYY25-19).

**Figure 1:**
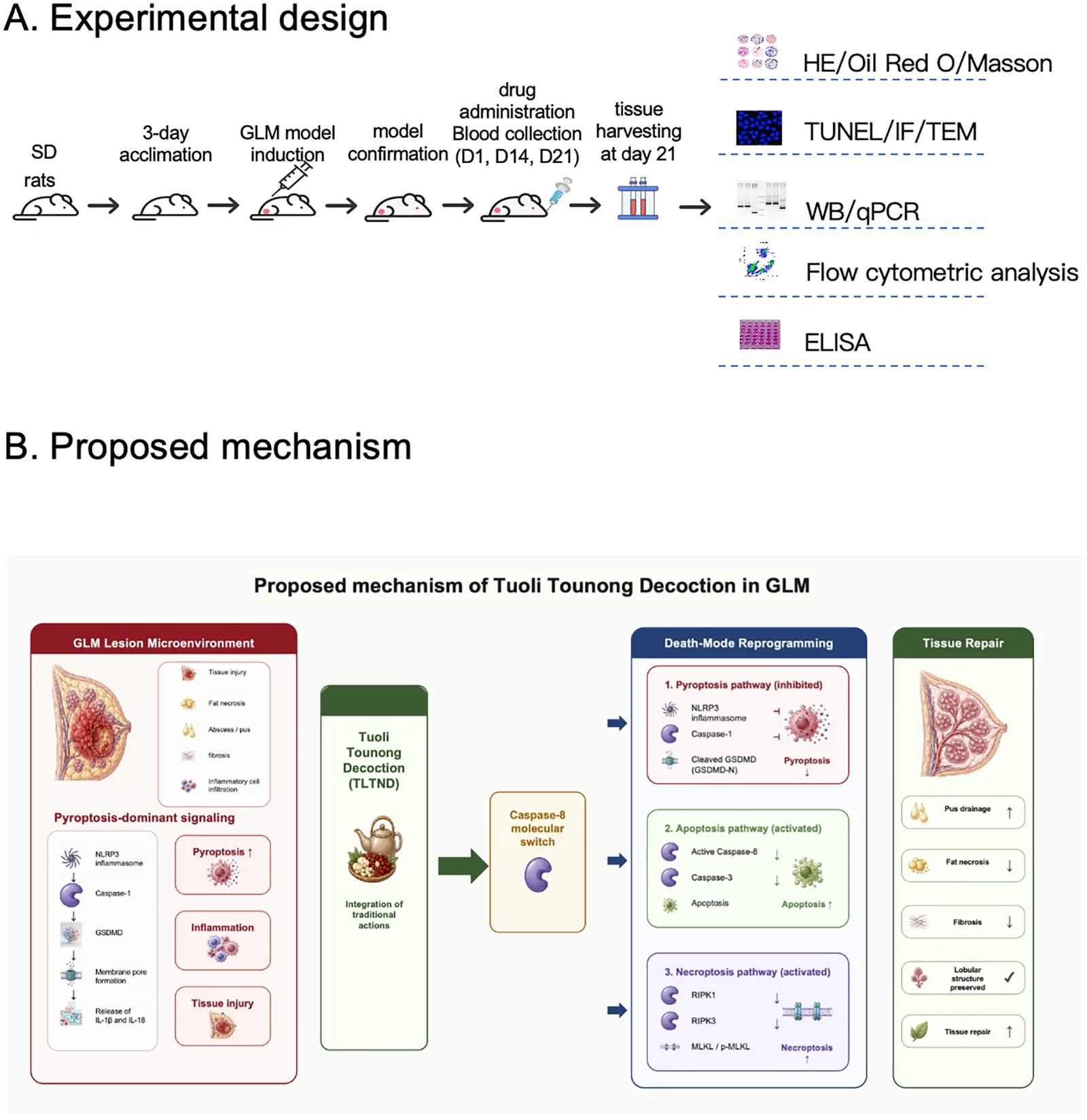
Experimental design and proposed mechanism. **(A)** Experimental workflow including GLM induction, treatment, blood collection, tissue harvesting, and subsequent analyses. Blood samples were collected on days 1, 14, and 21, and mammary tissues were harvested on day 21. **(B)** Proposed mechanism by which TTN-associated remodeling of a Caspase-8-associated PANoptosis-like cell death network, reprogramming to promote lesion clearance and tissue repair. GLM, granulomatous lobular mastitis; TTN, Tuoli Tounong Decoction; IF, immunofluorescence; TEM, transmission electron microscopy; WB, western blot; qPCR, quantitative real-time PCR; ELISA, enzyme-linked immunosorbent assay.

After acclimation, six rats were assigned to the control group and did not undergo model induction. The remaining 36 rats were used to establish the GLM model. After successful modeling, the modeled rats were randomly assigned using a random number table into the model, TF, TO, TTN, MP positive control, and C8i groups, with six rats in each group. Animal experimental design, randomization, outcome recording, and statistical analysis were described with reference to the ARRIVE 2.0 guidelines (21). The sample size was determined based on previous model-establishment experience and the ethical principle of reduction. No exclusion criteria were prespecified; deaths or severe non-experimental injuries, if any, were recorded and excluded. Body weight, mammary lesion length, histological images, and molecular data were collected and analyzed under standardized procedures. Blinding was applied during sample processing and microscopic image acquisition. Investigators responsible for tissue processing and microscopic image acquisition were blinded to group allocation. Group information was not revealed until completion of image acquisition and data recording. The overall experimental design and mechanistic framework are summarized in **Figure 1**.

### 2.2 Establishment of the GLM rat model

The GLM rat model was established according to a previously reported method (22). Human GLM lesion tissue used to prepare the inducing homogenate was obtained with approval from the Medical Ethics Committee of Dongzhimen Hospital, Beijing University of Chinese Medicine (approval No. 2025DZMEC-407-02). Written informed consent was obtained from all tissue donors. Except for the control group, rats were anesthetized, fixed, shaved, and disinfected, and a mixed inducing solution prepared from Freund’s adjuvant and human GLM lesion tissue homogenate was injected locally into the mammary gland at 0.1 mL per point, with four injection points. The control group received an equal volume of sterile saline at the same sites. Human granulomatous lobular mastitis (GLM) is characterized by lobule-centered non-caseating granulomatous inflammation, inflammatory cell infiltration, microabscess formation, fat necrosis, and local tissue destruction. The human GLM lesion tissue used for model induction was selected to provide pathological components relevant to clinical GLM. The rat model was designed to reproduce key pathological features of human GLM, including inflammatory infiltration, granuloma-like lesions, abscess-like changes, and tissue injury.

### 2.3 Drug preparation and intervention

The composition and dosing of the herbal interventions are provided in **Table 1**. All herbal decoction pieces were purchased from qualified pharmaceutical suppliers. Each herbal component was traceable by an individual batch number and complied with the quality standards specified in the Chinese Pharmacopoeia. Detailed information regarding the suppliers, batch numbers, and quality standards of individual herbal components is provided in **Supplementary Table 1**.

**Table 1:** Composition and dosing of herbal interventions.

Intervention group	Prescription composition	Individual herbal dosage	Administration dose
TF	Angelicae Sinensis Radix; Astragali Radix	Angelicae Sinensis Radix 10 g; Astragali Radix 15 g	2 g/kg/day
TO	Angelicae Dahuricae Radix; Gleditsiae Spina	Angelicae Dahuricae Radix 10 g; Gleditsiae Spina 10 g	2 g/kg/day
TTN	Ginseng Radix et Rhizoma; Atractylodis Macrocephalae Rhizoma; Vaccariae Semen; Angelicae Dahuricae Radix; Cimicifugae Rhizoma; Glycyrrhizae Radix et Rhizoma; Angelicae Sinensis Radix; Astragali Radix; Gleditsiae Spina; Citri Reticulatae Pericarpium Viride Preparatum	Ginseng Radix et Rhizoma 10 g; Atractylodis Macrocephalae Rhizoma 10 g; Vaccariae Semen 15 g; Angelicae Dahuricae Radix 10 g; Cimicifugae Rhizoma 10 g; Glycyrrhizae Radix et Rhizoma 6 g; Angelicae Sinensis Radix 10 g; Astragali Radix 15 g; Gleditsiae Spina 10 g; Citri Reticulatae Pericarpium Viride Preparatum 6 g	10.2 g/kg/day

Herbal doses are expressed as crude drug amounts. Administration doses were calculated by body surface area conversion between humans and rats. The different administration doses among TF, TO, and TTN groups resulted from differences in prescription composition and were not designed as a dose-gradient comparison.

Herbs were soaked in distilled water for 30 min according to the prescription ratio, decocted twice for 30 min and 15 min, respectively, and the combined filtrates were concentrated to the corresponding crude drug concentration. The prepared decoctions were stored at 4 °C and warmed to room temperature before administration.

Drug doses were determined by body surface area conversion between humans and rats. The dose selection was also supported by previous GLM-related experimental studies of Tuoli Tounong Decoction from our research group (23). The TF, TO, and TTN groups represented different therapeutic strategies rather than a dose-gradient design; therefore, differences in crude drug amounts resulted from differences in herbal composition and prescription characteristics. The doses of TF, TO, and TTN were 2 g/kg/day, 2 g/kg/day, and 10.2 g/kg/day, respectively, calculated as crude drug amounts. MP was administered at 4 mg/kg/day, and C8i was administered at 1 mg/kg/day. Except for C8i, which was administered by intraperitoneal injection, all treatments were given by gavage once daily for 21 consecutive days. The model group received an equal volume of saline.

### 2.4 General condition and local mammary observations

General condition and body weight were recorded daily. The maximum diameter of mammary lesions was measured every 2 days using vernier calipers, and local erythema, ulceration, pus exudation, and skin color changes were recorded.

### 2.5 Sample collection and processing

Blood samples were collected on days 1, 14, and 21. Serum was obtained after standing at room temperature followed by centrifugation at 3000 rpm for 15 min and stored at −80 °C for ELISA. At the endpoint, rats were sacrificed and mammary lesion tissues were rapidly collected. Some tissues were fixed in 4% paraformaldehyde, embedded in paraffin, and sectioned for H&E, Masson, TUNEL, and immunofluorescence staining. Fresh tissues were embedded in OCT and cryosectioned for Oil Red O staining. Some tissues were fixed in 2.5% glutaraldehyde for transmission electron microscopy. The remaining tissues were snap-frozen in liquid nitrogen and stored at −80 °C for qPCR and Western blotting. Fresh mammary tissues were also used to prepare single-cell suspensions for flow cytometry.

### 2.6 Histopathological staining

Paraffin sections were deparaffinized, rehydrated, and subjected to H&E and Masson’s trichrome staining. H&E staining was used to evaluate mammary tissue architecture, inflammatory cell infiltration, granuloma formation, and abscess-like changes. Masson staining was used to assess collagen deposition, fiber arrangement, and fibrosis-like changes. Fresh mammary tissues embedded in OCT were cryosectioned at 5 µm and stained with Oil Red O to assess lipid distribution, adipose tissue destruction, fat necrosis, and foam cell-like changes. All sections were observed and imaged using a light microscope.

### 2.7 TUNEL staining

TUNEL staining was performed to detect apoptosis in mammary tissues. Paraffin sections were deparaffinized, rehydrated, repaired with proteinase K, incubated with TUNEL reaction solution in the dark, and counterstained with DAPI. Multiple fields were randomly selected from each section and imaged under identical exposure settings. TUNEL-positive area intensity was quantified using ImageJ.

### 2.8 Multiplex immunofluorescence staining and confocal microscopy

Multiplex immunofluorescence staining was performed to detect GSDMD, p-MLKL, and macrophage-related markers F4/80 and CD11c in mammary tissues. Paraffin sections were deparaffinized, rehydrated, subjected to antigen retrieval, and blocked, followed by incubation with primary antibodies overnight at 4 °C. Primary antibodies included anti-F4/80 (1:1000), anti-CD11c (1:50), anti-p-MLKL (1:100), and anti-GSDMD (1:100). The next day, sections were incubated with corresponding fluorescent secondary antibodies (1:800) in the dark and counterstained with DAPI. All antibodies were purchased from Beijing Huaxingbio Gene Technology Co., Ltd. (Huaxingbio, Beijing, China). Images were acquired using a laser confocal microscope under identical acquisition parameters, and mean fluorescence intensity of each channel was measured using ImageJ.

### 2.9 Transmission electron microscopy

Approximately 1 mm³ mammary lesion tissue was immediately fixed in 2.5% glutaraldehyde. Samples were post-fixed with osmium tetroxide, dehydrated through graded ethanol, embedded in resin, ultrathin-sectioned, and stained with uranyl acetate and lead citrate. Macrophage ultrastructure was observed using transmission electron microscopy at 12, 000× and 20, 000×. Cell membrane integrity, membrane pore-like changes, ballooning protrusions, mitochondrial swelling, chromatin margination, nuclear fragmentation, and apoptotic body-like structures were evaluated.

### 2.10 qPCR analysis

Total RNA was extracted from mammary tissues using TRIzol combined with column purification. RNA concentration and purity were assessed, and RNA was reverse-transcribed into cDNA. qPCR was used to detect *Casp8* mRNA expression and *Casp8* C362S-associated amplification signals. The *Casp8* primer sequences were: forward, AGGGAACAGGAAGTGAGCCC; reverse, GTAGGAGAGCTGTAACCTGTCG. The *Casp8* C362S-associated amplification primer sequences were: forward, CTACGGAACGGATGGGAAGG; reverse, AGTGTGAAGATGGGCTGTGG. GAPDH was used as the internal reference gene, and relative expression was calculated using the 2^-DeltaDeltaCt method.

### 2.11 Western blotting

Mammary tissues were lysed in RIPA buffer containing protease and phosphatase inhibitors, and total protein concentration was determined using the BCA method. Equal amounts of protein were separated by SDS-PAGE and transferred to PVDF membranes. Phosphorylated proteins were blocked with 5% BSA, whereas non-phosphorylated proteins were blocked with 5% non-fat milk. Membranes were then incubated with primary antibodies overnight at 4 °C. Primary antibodies included Caspase-8, cleaved Caspase-8, NLRP3, Caspase-1, cleaved Caspase-1, GSDMD, cleaved GSDMD, Caspase-3, cleaved Caspase-3, MLKL, p-MLKL, and β-actin, all at 1:1000 dilution. The next day, membranes were incubated with HRP-conjugated secondary antibodies (1:10000), and bands were visualized using enhanced chemiluminescence. All antibodies were purchased from Beijing Huaxingbio Gene Technology Co., Ltd. β-actin was used as the loading control, and band intensities were quantified using ImageJ.

### 2.12 Flow cytometry

Fresh mammary tissues were minced and digested with collagenase and DNase I to prepare single-cell suspensions. Suspensions were filtered, subjected to red blood cell lysis, washed with PBS, and incubated with fluorescent antibodies in the dark. The flow cytometry antibodies included CD43-PC7, CD68-PE, CD11c-FITC, CD11b-ECD, and CD86-APC. CD43 and CD68 were used to assist in identifying mammary immune/macrophage-related populations, and CD86, CD11c, and CD11b expression was further analyzed to evaluate macrophage/myeloid cell-related subsets and phenotypic changes in mammary tissues. Flow cytometry data were analyzed using FlowJo after excluding debris and doublets, and both the proportions and absolute numbers of CD86-, CD11c-, and CD11b-positive cells were assessed.

### 2.13 ELISA

Serum IL-1β and IL-18 levels were measured using ELISA kits purchased from Huangshi INSELISA Biotechnology Co., Ltd. (Huangshi, China; IL-18 Cat. No. INS-R0013; IL-1β Cat. No. INS-R0015) according to the manufacturer’s instructions. Absorbance was read at 450 nm using a microplate reader, and cytokine concentrations were calculated from standard curves.

### 2.14 Statistical analysis

**Figure 2:**
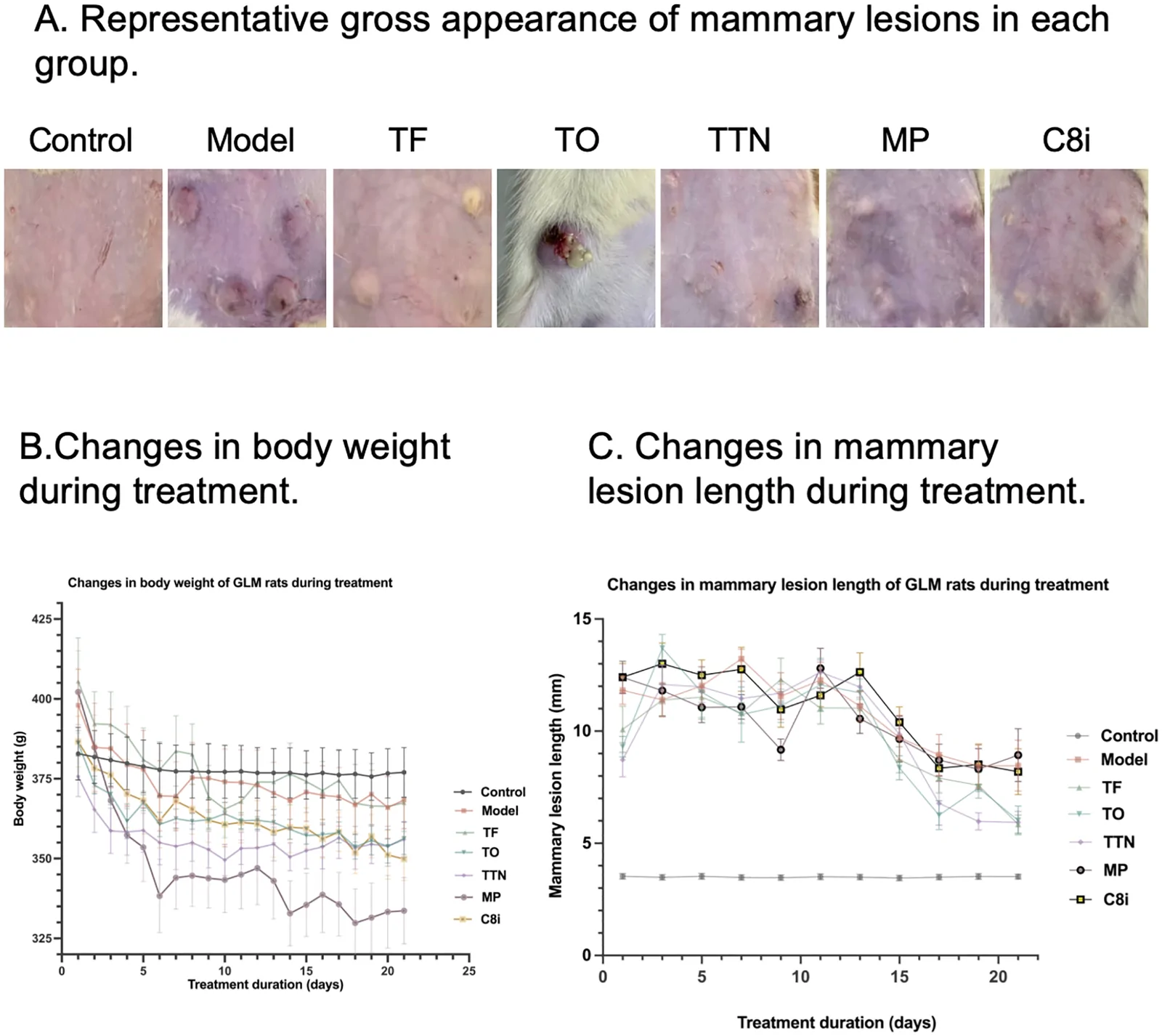
General condition and local mammary changes in GLM rats during the intervention period. **(A)** Representative gross appearance of mammary lesions in each group. **(B)** Changes in body weight during treatment. **(C)** Changes in mammary lesion length during treatment. Data are presented as mean ± SEM (n=6 per group). Statistical analysis was performed using two-way repeated-measures ANOVA followed by Tukey’s multiple comparisons test. Control, normal control group; Model, GLM model group; TF, supporting method group; TO, draining method group; TTN, Tuoli Tou Nong Decoction group; MP, methylprednisolone group; C8i, Caspase-8 inhibitor group.

All data were analyzed using GraphPad Prism 11. Quantitative data are presented as mean ± SD unless otherwise indicated. Data in **Figure 2**, including body weight and mammary lesion length, are presented as mean ± SEM. One-way analysis of variance was used for comparisons among multiple groups, and two-way repeated-measures ANOVA was used for time-course parameters, including body weight and mammary lesion length. Tukey’s test was used for *post hoc* comparisons when variances were homogeneous, and Dunnett’s T3 test was used when variances were unequal. P < 0.05 was considered statistically significant.

## 3 Results

### 3.1 General condition of rats

Body weight, mammary gland length, and local mammary appearance were dynamically monitored for 21 days (**Figure 2**). Body weight remained generally stable in the control group. In the model group, body weight decreased during the early stage and then gradually stabilized. The TF, TO, and TTN groups showed early body weight loss followed by recovery around day 10, whereas body weight loss was more obvious in the MP and C8i groups.

Mammary gland length remained stable in the control group but gradually increased in the model group, with obvious local mass formation. Compared with the model group, mammary gland length decreased in the TF and TO groups and decreased most markedly in the TTN group. Mammary gland length in the MP group decreased initially and then increased, whereas the C8i group showed some improvement compared with the model group but less than the herbal intervention groups.

Local mammary observation showed that the control group had a smooth mammary surface without obvious erythema, ulceration, or pus exudation. The model group showed marked mammary enlargement, multiple masses, and local purplish-red discoloration. The TF group was characterized mainly by pus accumulation without obvious rupture. The TO group showed more unobstructed pus drainage and reduced mammary volume. The TTN group showed sufficient pus drainage, markedly reduced mammary volume, and skin color closer to normal. The MP group showed restricted pus drainage and diffuse swelling, whereas the C8i group still exhibited obvious local inflammation. Overall, TTN showed advantages in promoting pus drainage, reducing mammary mass size, and restoring local appearance.

### 3.2 Integrated analysis of mammary histopathology, lipid injury, and fibrosis

#### 3.2.1 H&E staining shows inflammatory injury and granuloma formation

H&E staining showed intact mammary lobular structure, regular acinar and ductal arrangement, and no obvious inflammatory infiltration, abscess formation, or tissue necrosis in the control group. In the model group, lobular and ductal structures were markedly damaged, with abundant inflammatory cell infiltration, granuloma-like structures, and microabscess formation, indicating obvious inflammatory injury in mammary tissues of GLM model rats.

**Figure 3:**
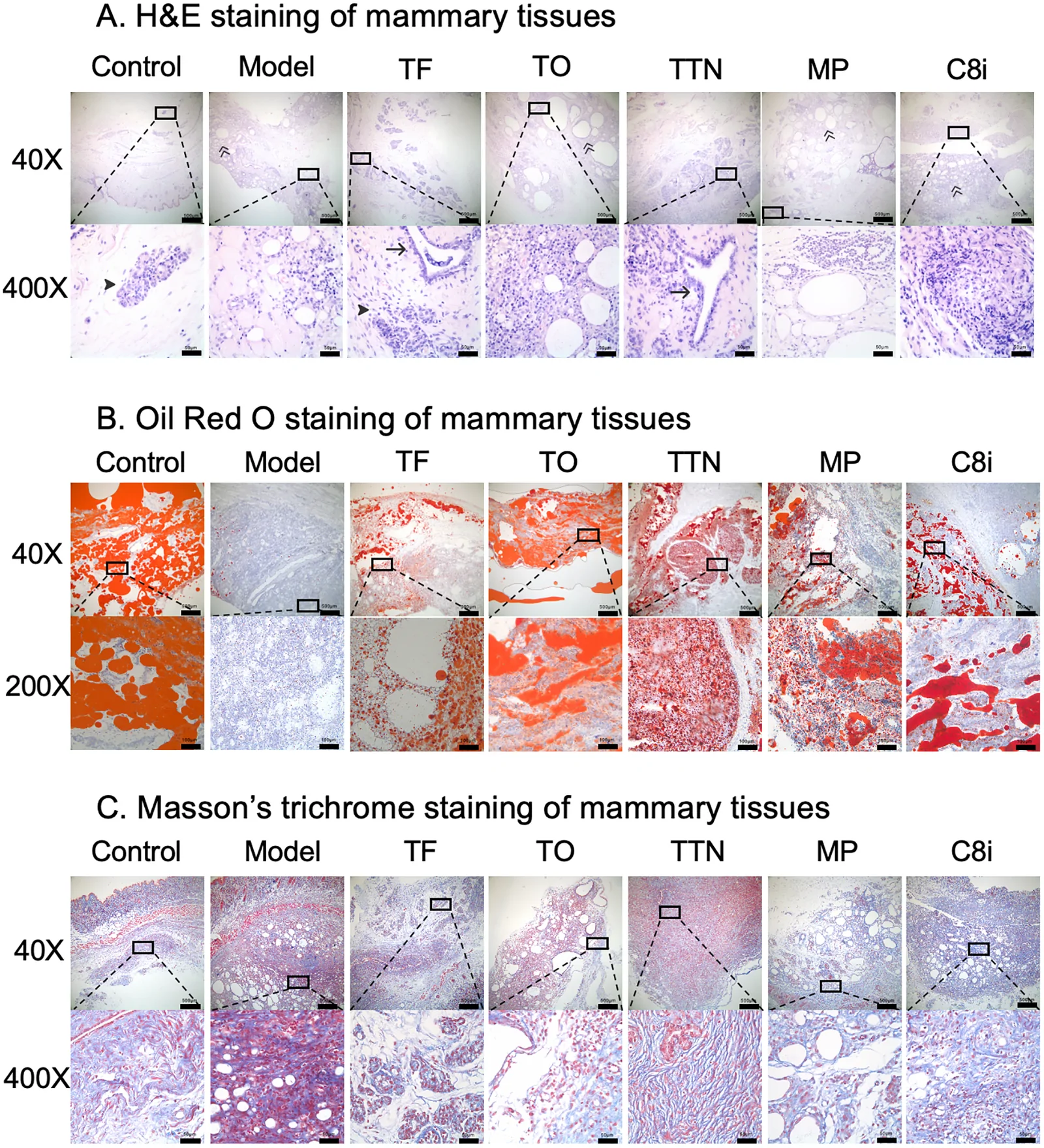
Histopathological, lipid injury, and fibrosis changes in mammary tissues. **(A)** H&E staining showing inflammatory cell infiltration, granuloma-like structures, and abscess-like lesions. **(B)** Oil Red O staining showing lipid distribution, adipose tissue destruction, fat necrosis, and foam cell-like changes. **(C)** Masson staining showing collagen deposition and fibrosis-like remodeling.

Compared with the model group, each intervention group showed varying degrees of pathological improvement. The TF group retained relatively clear lobular and ductal outlines, with a smaller area of tissue destruction. The TO group showed larger abscess cavities while retaining some lobular and ductal structures. The TTN group showed better preservation of lobular and ductal architecture, reduced inflammatory infiltration, and milder tissue destruction. In contrast, the MP and C8i groups still showed residual abscesses, inflammatory infiltration, and mammary structural destruction, suggesting insufficient tissue repair. Representative images are shown in **Figure 3**.

#### 3.2.2 Oil Red O staining shows adipose tissue destruction and lipid-related inflammation

Oil Red O staining showed intact adipocyte morphology and relatively uniform lipid distribution in the control group, with no obvious fat necrosis or abscess-like changes. The model group showed marked adipose tissue destruction, a substantial reduction in normal fat structure, fat necrosis, abscess formation, lipophilic inflammatory infiltration, and foam cell-like changes, indicating prominent fat injury and lipid-related inflammation in GLM lesions.

Compared with the model group, the TF, TO, and TTN groups showed reduced adipose destruction, with relatively more residual fat structures and fewer lipophilic inflammatory cells and foam cell-like changes. The TTN group showed milder fat necrosis and abscess-like changes and relatively preserved adipose tissue architecture. The MP and C8i groups still showed obvious fat necrosis, abscess-like changes, and foam cell accumulation, indicating limited improvement in GLM-associated fat injury. Representative images are shown in **Figure 3**.

#### 3.2.3 Masson staining shows collagen deposition and fibrosis-like changes

Masson staining showed intact mammary tissue architecture with sparse and regularly arranged collagen fibers in the control group. In the model group, mammary tissue architecture was markedly disrupted, and abundant blue-green collagen fibers were deposited around abscesses and necrotic foci, with disordered and dense arrangement, indicating obvious fibrosis-like changes.

Compared with the model group, collagen deposition and fibrosis were reduced in all intervention groups. The TF group showed relatively preserved lobular structure, reduced collagen proliferation, and looser fiber arrangement. The TO group showed large cavity-like structures, reduced collagen deposition, and partially preserved lobular and ductal structures. The TTN group showed better preservation of lobules and ducts, reduced collagen deposition, relatively uniform fiber distribution, and milder inflammatory infiltration and fibrosis-like changes. The MP and C8i groups still showed varying degrees of collagen proliferation, local fibrosis-like changes, and residual inflammatory cells. Representative images are shown in **Figure 3**.

### 3.3 Cell death-related signals in mammary tissues

#### 3.3.1 TUNEL staining shows apoptosis-related cell death signals in GLM lesions

**Figure 4:**
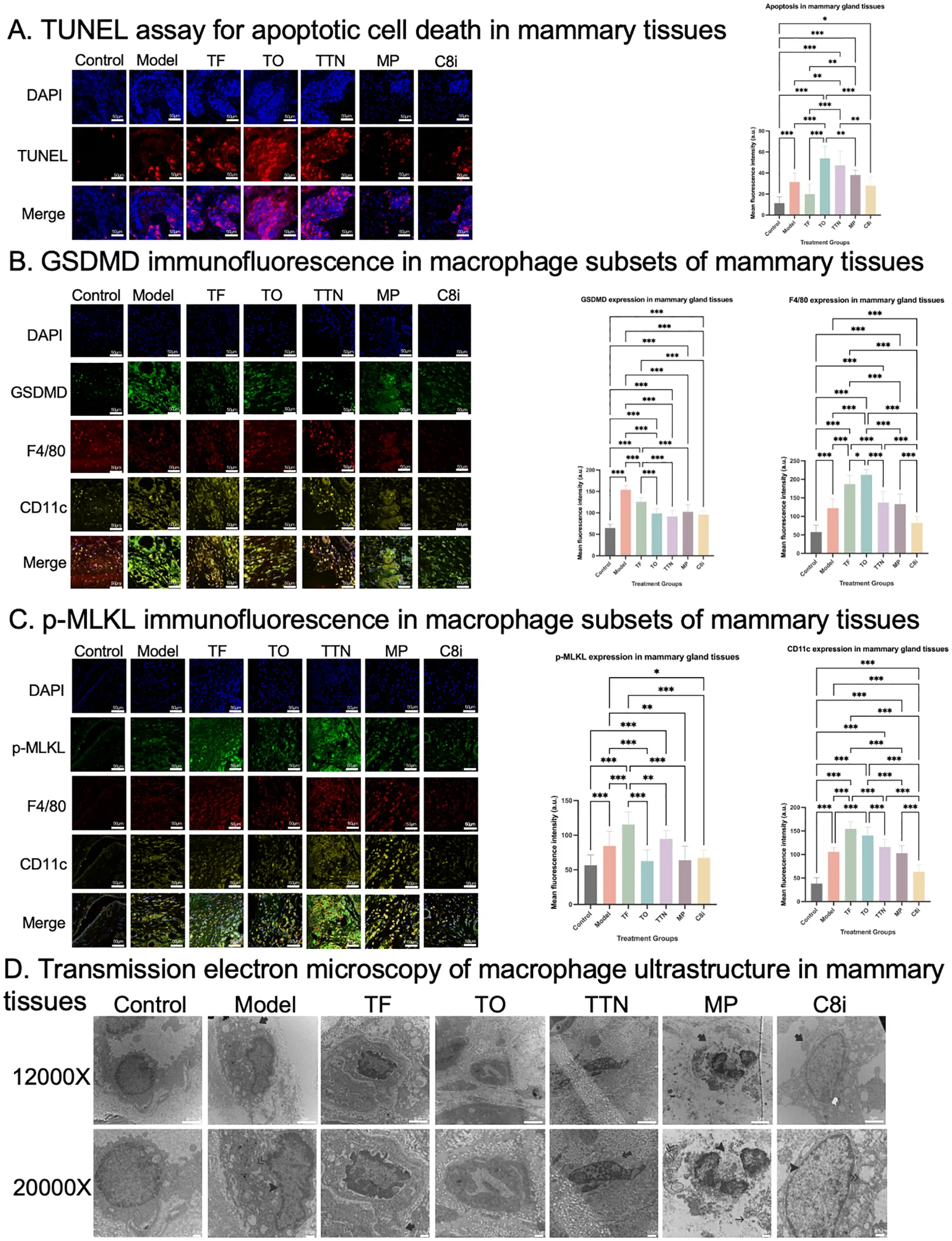
Cell death-related signals and ultrastructural changes in mammary tissues. **(A)** TUNEL staining and quantification of apoptosis-related cell death. **(B)** Immunofluorescence analysis of GSDMD, F4/80, and CD11c. **(C)** Immunofluorescence analysis of p-MLKL, F4/80, and CD11c. **(D)** TEM images showing macrophage ultrastructural changes. Data are presented as mean ± SD. Scale bars are indicated in the images. *p<0.05, **p<0.01, ***p<0.001.

TUNEL staining showed only a small number of scattered weakly positive signals in the control group. Compared with the control group, TUNEL-positive cells increased slightly in the model group, although the overall signal remained weak. Compared with the model group, TUNEL positivity increased in the TF group. The TO group showed the strongest TUNEL signal with numerous positive cells, mainly around abscesses and inflammatory lesions. The TTN group also showed enhanced TUNEL positivity, although the overall signal was weaker than that in the TO group. The MP group showed intermediate TUNEL positivity, whereas the C8i group showed weaker signals. Combined with cleaved Caspase-3 expression and TEM findings, these results suggest that TO and TTN enhanced apoptosis-related cell death signals in GLM lesions to different degrees, with TO showing more prominent apoptosis-related changes. Representative images and quantitative results are shown in **Figure 4**.

#### 3.3.2 Multiplex immunofluorescence shows pyroptosis, necroptosis, and macrophage-related signals

To further evaluate cell death-related signals in GLM lesions, multiplex immunofluorescence was used to detect GSDMD, p-MLKL, and macrophage-related markers F4/80 and CD11c, and mean fluorescence intensity was quantified for each channel. Compared with the control group, the model group showed markedly increased GSDMD mean fluorescence intensity and membrane-associated signal accumulation, suggesting enhanced pyroptosis-related signaling in GLM lesions. Compared with the model group, GSDMD fluorescence intensity decreased to varying degrees in the TF, TO, and TTN groups, especially in the TO and TTN groups. Because immunofluorescence detected total GSDMD, membrane accumulation of GSDMD was used mainly as supportive spatial evidence for pyroptosis-related localization changes, and the execution of pyroptosis was further evaluated together with cleaved GSDMD expression by Western blotting.

p-MLKL immunofluorescence showed that the model group had higher mean fluorescence intensity than the control group, suggesting activation of necroptosis-related signaling in GLM lesions. Among the intervention groups, p-MLKL fluorescence intensity was higher in the TF and TTN groups, especially in the TTN group, whereas p-MLKL signals were relatively lower in the TO, MP, and C8i groups. F4/80 and CD11c fluorescence intensity analysis showed increased macrophage-related signals in the model group compared with the control group. After intervention, F4/80 and CD11c signals varied among groups, indicating differential effects of different interventions on local macrophage infiltration and related phenotypes in GLM lesions. Representative images and quantitative results are shown in **Figure 4**.

### 3.4 TEM reveals macrophage ultrastructure and programmed cell death features

TEM showed intact macrophage morphology, continuous cell membranes, and clear nuclei and organelles in the control group. In the model group, macrophages showed cell swelling, impaired membrane continuity, membrane pore-like changes, ballooning protrusions, and mitochondrial swelling, suggesting pyroptosis-related ultrastructural features. The TF and C8i groups were characterized mainly by cytoplasmic and organelle swelling and impaired membrane integrity, compatible with necroptosis-related morphological features. The TO and TTN groups showed chromatin margination, nuclear fragmentation, apoptotic body-like structures, and relatively intact membranes, suggesting prominent apoptosis-related alterations. The MP group still exhibited membrane injury, ballooning protrusions, and mitochondrial swelling, consistent with pyroptosis-related morphology. Together with TUNEL and immunofluorescence findings, these results suggest that TTN may participate in local inflammatory clearance and tissue repair in GLM by suppressing excessive pyroptosis and promoting apoptosis- and necroptosis-associated lesion resolution. Representative images are shown in **Figure 4**.

### 3.5 qPCR shows changes in mammary Casp8 expression and C362S-associated amplification signals

**Figure 5:**
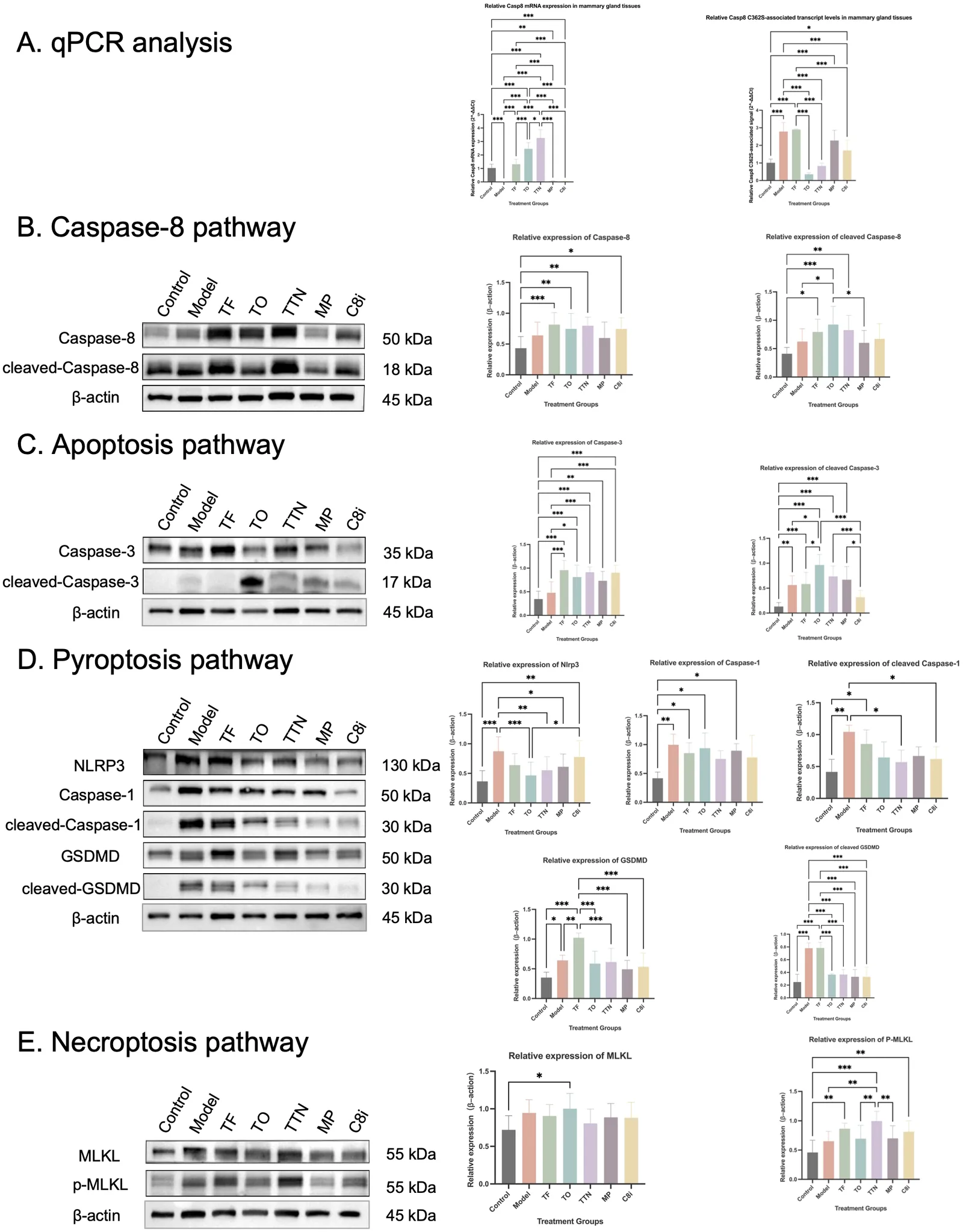
qPCR and Western blot analysis of Caspase-8 and cell death-related signaling. **(A)** qPCR analysis of Casp8 mRNA expression and Casp8 C362S-associated amplification signals. **(B)** Caspase-8 and cleaved Caspase-8. **(C)** Apoptosis-related proteins, including Caspase-3 and cleaved Caspase-3. **(D)** Pyroptosis-related proteins, including NLRP3, Caspase-1, cleaved Caspase-1, GSDMD, and cleaved GSDMD. **(E)** Necroptosis-related proteins, including MLKL and p-MLKL. Data are presented as mean ± SD. *p<0.05, **p<0.01, ***p<0.001.

qPCR showed significant differences in Casp8 mRNA expression and Casp8 C362S-associated amplification signals among groups. Compared with the control group, Casp8 mRNA expression was decreased in the model group. Casp8 mRNA expression was increased in the TF, TO, and TTN groups, with the highest expression in the TTN group, whereas the MP and C8i groups remained at relatively low levels. Casp8 C362S-associated amplification signals were higher in the model and TF groups, and also remained relatively high in the MP and C8i groups. The TO group showed the lowest C362S-associated signal, and the TTN group showed relatively low levels. Quantitative results and statistical differences are shown in **Figure 5**.

### 3.6 Western blotting shows changes in Caspase-8 and apoptosis-, pyroptosis-, and necroptosis-related proteins

Western blotting showed differences in Caspase-8 and programmed cell death-related proteins among groups. Compared with the control group, Caspase-8 expression tended to increase in the modeled groups, with more obvious increases in the TF, TO, TTN, and C8i groups. Cleaved Caspase-8 was markedly increased in the TF, TO, and TTN groups, whereas its expression was relatively lower in the MP and C8i groups.

Analysis of apoptosis-related proteins showed that total Caspase-3 was highest in the TF group, and other intervention groups also showed varying degrees of increase compared with the model group. Cleaved Caspase-3 was most prominent in the TO group, and increased in the TTN and MP groups, although to a lesser extent than in the TO group. Analysis of pyroptosis-related proteins showed that NLRP3, cleaved Caspase-1, and cleaved GSDMD were markedly increased in the model group, indicating activation of pyroptosis-related pathways in GLM lesions. Compared with the model group, NLRP3, cleaved Caspase-1, and cleaved GSDMD were reduced to varying degrees in the TO, TTN, MP, and C8i groups, with a particularly obvious reduction in cleaved GSDMD in the TTN group. In the TF group, GSDMD and cleaved GSDMD remained relatively high.

Analysis of necroptosis-related proteins showed that total MLKL varied only slightly among groups, whereas p-MLKL showed more obvious changes. p-MLKL expression was highest in the TTN group, followed by the TF group. The model and TO groups showed intermediate levels, whereas the MP and C8i groups showed relatively weak expression.

Overall, the model group was characterized mainly by activation of pyroptosis-related proteins. The TO group was characterized by increased cleaved Caspase-8 and cleaved Caspase-3. The TF group showed relatively high GSDMD and p-MLKL expression. The TTN group showed increased cleaved Caspase-8, cleaved Caspase-3, and p-MLKL, accompanied by reduced cleaved GSDMD. These findings suggest that different interventions differentially regulate the Caspase-8 pathway and apoptosis-, pyroptosis-, and necroptosis-related proteins, and that TTN may be associated with a shift in cell death-related molecular profiles from excessive pyroptosis-related signals toward apoptosis- and necroptosis-associated signals. Quantitative results and statistical differences are shown in **Figure 5**.

### 3.7 Flow cytometry shows changes in macrophage/myeloid cell-related subsets in mammary tissues

Flow cytometry showed differences in both the proportions and absolute numbers of CD86-positive, CD11c-positive, and CD11b-positive cells among groups. Compared with the control group, the model group showed an increased proportion of CD86-positive cells. After intervention, CD86-positive cells were highest in the TO group, followed by the TF and TTN groups. Absolute numbers showed a similar trend.

CD11c-positive cell analysis showed that both the proportion and absolute number of CD11c-positive cells were highest in the TO group, followed by the TTN and TF groups. The model group also showed some increase, whereas the MP and C8i groups showed lower expression.

CD11b-positive cell analysis showed increased CD11b-positive cells in the model group. After intervention, CD11b-positive cells remained high, with the highest level in the TO group, followed by the TF and TTN groups, whereas the MP and C8i groups showed lower expression.

**Figure 6:**
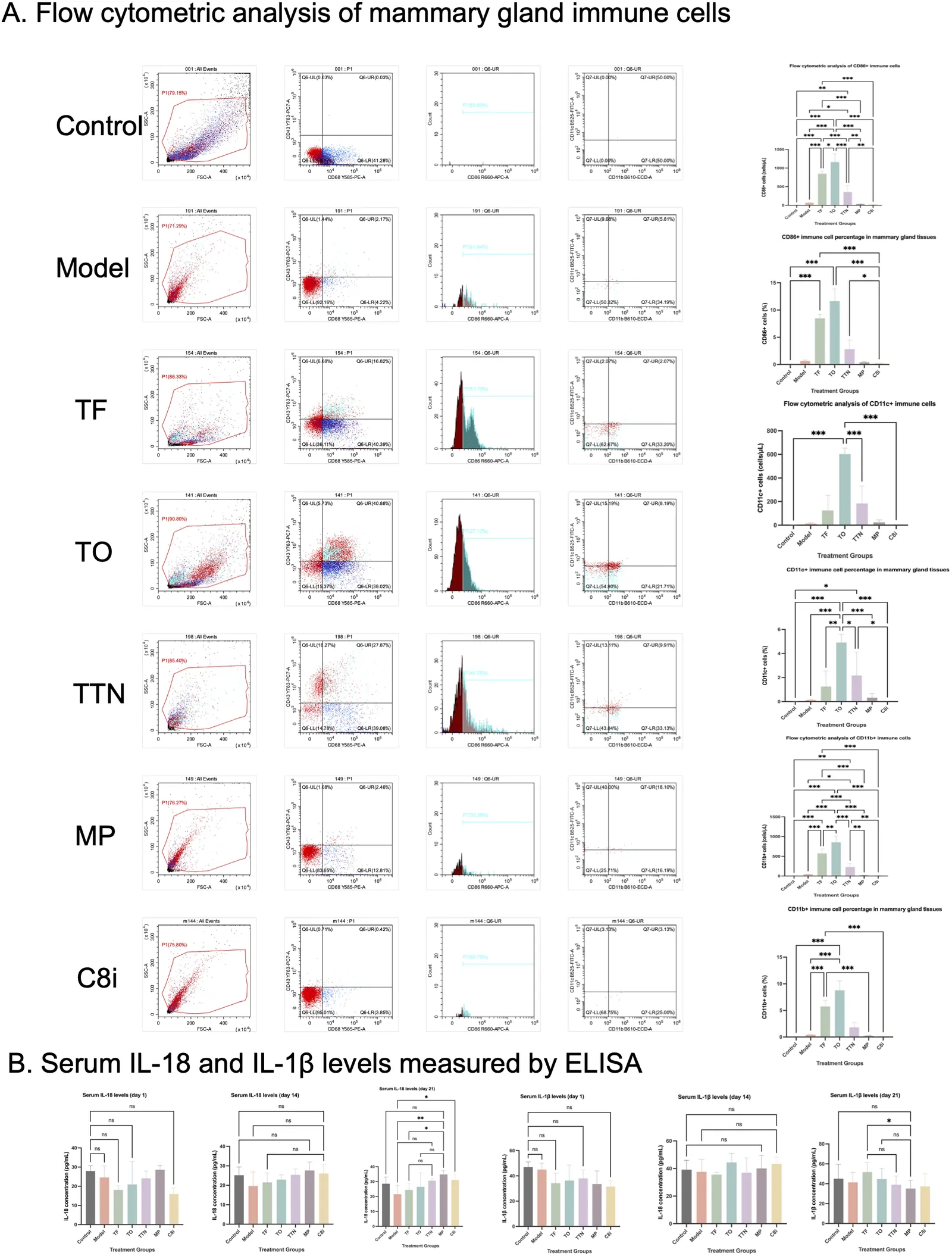
Local immune cell remodeling and serum inflammatory cytokine levels. **(A)** Flow cytometry gating strategy and quantification of CD86-, CD11c-, and CD11b-positive mammary macrophage/myeloid cell-related subsets, expressed as proportions and absolute numbers. **(B)** Serum IL-1β and IL-18 levels measured by ELISA on days 1, 14, and 21. Data are presented as mean ± SD. *p<0.05, **p<0.01, ***p<0.001, ns: no significant difference.

Overall, CD86-positive, CD11c-positive, and CD11b-positive myeloid immune cells were significantly remodeled in mammary tissues of GLM model rats. Different interventions differentially regulated local immune cell activation and recruitment. The TO group showed the strongest immune cell recruitment, followed by the TF group, whereas the TTN group showed a relatively balanced immune regulatory profile. The MP and C8i groups showed prominent immunosuppressive patterns. Quantitative results and statistical differences are shown in **Figure 6**.

### 3.8 Dynamic changes in serum IL-18 and IL-1β levels

ELISA was used to measure serum IL-18 and IL-1β levels on days 1, 14, and 21. No obvious differences in IL-18 or IL-1β were observed among groups on day 1. On day 14 after modeling, IL-18 and IL-1β levels were slightly increased in the modeled groups, but no statistically significant differences were observed among groups. On day 21, serum IL-18 and IL-1β levels showed no obvious fluctuations, and between-group comparisons remained non-significant. These results suggest that GLM induction induced a tendency toward increased peripheral IL-18 and IL-1β, but different interventions had no obvious effect on serum inflammatory cytokine levels. Quantitative results and statistical differences are shown in **Figure 6**.

## 4 Discussion

GLM is a chronic inflammatory disease primarily involving mammary lobules. Its development is closely associated with ductal epithelial injury, extravasation of mammary secretions, macrophage and lymphocyte recruitment, and granulomatous reaction formation (24). In recent years, GLM has been increasingly considered not as a single inflammatory disease, but as a complex disorder involving local immune microenvironment imbalance, inflammatory cell death, and abnormal tissue repair (24, 25). In this study, the effects of TTN on mammary lesions in GLM rats were evaluated from multiple perspectives, including histopathology, cell death-related signals, the Caspase-8 pathway, macrophage-related subsets, and serum inflammatory cytokines. The results showed that TTN not only alleviated inflammatory injury, fat destruction, and collagen deposition in mammary tissues, but also induced differential changes in Caspase-8 and apoptosis-, pyroptosis-, and necroptosis-related signals. These findings suggest that TTN does not simply suppress inflammation in GLM, but may promote lesion clearance and tissue repair by regulating local immune cell infiltration and programmed cell death modes.

### 4.1 TTN improves local pathological injury and promotes lesion resolution in GLM

Pathologically, GLM is commonly characterized by lobule-centered non-caseating granulomas, inflammatory cell infiltration, microabscess formation, fat necrosis, and local tissue destruction (2). From the perspective of lesion evolution, GLM is not merely inflammatory cell infiltration, but a dynamic process involving inflammatory injury, lipid destruction, granuloma formation, and fibrosis-like repair. Clinically, breast masses, abscesses, ulceration, sinus tract formation, and scar repair may occur sequentially or overlap (2, 26–28). In addition, after mammary duct epithelial injury or increased ductal permeability, milk and ductal secretions can extravasate into the interlobular stroma, acting as local antigenic stimuli that induce T-cell and macrophage infiltration and delayed-type hypersensitivity-like local immune responses, further promoting granuloma formation and lesion persistence (2, 26, 29).

Against this pathological background, H&E, Oil Red O, and Masson staining in the model group showed inflammatory cell infiltration, destruction of lobular and ductal structures, fat necrosis, abscess formation, and collagen deposition, indicating that the model lesions recapitulated the coexistence of inflammatory destruction, lipid injury, and fibrosis-like repair in GLM. Traditional herbal formulas have multicomponent, multitarget, and multipathway pharmacological characteristics. Previous studies suggest that traditional Chinese medicine may exert comprehensive effects in GLM by regulating local inflammatory responses, immune cell function, and tissue repair (12–15). Compared with the model group, the improvements observed in the herbal intervention groups were not limited to reduced inflammatory cells, but more clearly reflected promotion of lesion resolution. The TF group showed relatively well-preserved lobular and ductal structures, suggesting that it may help restrict inflammatory destruction and maintain local structural integrity. The TO group showed more obvious abscess opening and pus discharge, suggesting that it may promote the transition of closed lesions toward open clearance. The TTN group combined structural protection and lesion discharge, with reduced fat necrosis and collagen deposition, suggesting that it may promote the transition of GLM lesions from inflammatory destruction toward clearance and repair.

In contrast, although MP exerted certain anti-inflammatory effects, residual lesions, fat necrosis, and collagen deposition were still observed histologically, suggesting that local immunosuppression alone is insufficient to fully promote GLM lesion resolution. If antigenic contents, necrotic tissue, abscess material, and inflammatory dying cells in the interlobular stroma are not effectively cleared, residual lesions may continue to stimulate local immune responses, delay tissue repair, and increase the risk of recurrence or new lesion formation even when inflammation is temporarily reduced (30, 31). Therefore, the pathological findings suggest that TTN improves local pathological injury in GLM through integrated effects on lesion clearance, tissue repair, and mammary structural protection, providing morphological evidence for further exploration of its regulation of cell death.

### 4.2 TTN promotes GLM lesion resolution through remodeling of Caspase-8-associated PANoptosis-like cell death signaling

This study showed that pyroptosis-related changes were prominent in GLM lesions of the model group, including increased NLRP3, cleaved Caspase-1, and cleaved GSDMD expression, enhanced membrane-associated GSDMD signals, and ultrastructural features such as membrane pore-like changes, ballooning protrusions, and mitochondrial swelling. After herbal interventions, cell death-related signals were differentially remodeled. The TF group showed relatively preserved GSDMD-related signals and increased p-MLKL expression. The TO group showed the strongest TUNEL positivity, increased cleaved Caspase-8 and cleaved Caspase-3 expression, and ultrastructural features such as chromatin margination, nuclear fragmentation, and apoptotic body-like structures, suggesting enhanced apoptosis-associated characteristics. The TTN group showed increased TUNEL positivity, cleaved Caspase-3, and p-MLKL expression, together with reduced GSDMD membrane accumulation and cleaved GSDMD levels, suggesting that TTN may attenuate excessive pyroptosis-related inflammatory signals while promoting Caspase-8-associated PANoptosis-like cell death signal remodeling.

Programmed cell death has distinct roles in inflammatory and immune-mediated diseases. Pyroptosis is a GSDMD-mediated lytic inflammatory form of cell death that amplifies local inflammatory responses through membrane pore formation, cell swelling, and inflammatory mediator release (8). Previous studies reported increased cleaved GSDMD and cleaved Caspase-1 expression and pyroptosis-like ultrastructural features in GLM tissues, suggesting that pyroptosis contributes to inflammatory amplification and tissue destruction in GLM (6). In contrast, apoptosis is generally a relatively low-inflammatory and regulated form of cell death. Apoptotic cells can be cleared by macrophage efferocytosis, which has been reported to contribute to limiting secondary inflammation and tissue repair (9, 32). In GLM lesions, where fat destruction is common, failure to clear dead or damaged adipocytes may generate persistent lipid stimulation, induce macrophage accumulation, and sustain local inflammation (33). Conversely, controlled death and macrophage-mediated clearance of injured adipocytes may reduce secondary necrosis, limit inflammation spread, and promote tissue remodeling (9, 34). In this study, Oil Red O staining showed reduced fat necrosis and foam cell-like changes in herbal intervention groups. Together with enhanced TUNEL signals and attenuated pathological injury, these findings suggest that herbal interventions may modulate cell death-related processes and reduce inflammatory tissue damage, thereby contributing to GLM lesion resolution.

Necroptosis is mainly associated with the RIPK3/MLKL pathway and has context-dependent effects. It can contribute to inflammation and tissue injury under certain conditions, while also being involved in regulated tissue responses and remodeling processes depending on the biological context (10, 35). Therefore, increased p-MLKL in the TTN group should not be interpreted simply as aggravated tissue injury, but rather in the context of reduced pyroptosis, enhanced apoptosis, and improved pathology. This suggests that necroptosis-related signals may participate in lesion boundary remodeling, dying-cell processing, and activation of repair responses.

The concept of PANoptosis proposes that pyroptosis, apoptosis, and necroptosis are not isolated events, but may be cross-regulated in inflammatory, infectious, or tissue-injury settings, suggesting the existence of an integrated programmed cell death regulatory network (7, 36, 37). Thus, in GLM, a disease characterized by chronic inflammation, fat necrosis, abscess formation, and repair remodeling, a single death pathway may be insufficient to explain lesion persistence and resolution. Targeting PANoptosis-like regulatory pathways involving inflammasomes, RIPK, and caspase pathways has been suggested as a potential therapeutic strategy for inflammatory, infectious, and malignant diseases (38). This provides a theoretical basis for understanding GLM treatment from the perspective of a PANoptosis-like cell death network.

### 4.3 Caspase-8 may be a key node in TTN-associated PANoptosis-like cell death remodeling

Caspase-8 was initially regarded mainly as an initiator caspase in the extrinsic apoptosis pathway, activating the downstream Caspase-3/7 cascade after death receptor signaling and inducing apoptosis (39). Subsequent studies showed that Caspase-8 is not merely an upstream apoptosis molecule, but a critical intersection between cell death and inflammatory regulation. It promotes Caspase-3-mediated apoptosis, regulates RIPK1/RIPK3/MLKL-associated necroptotic signaling, and participates in inflammasome-associated signaling and IL-1β processing (40–43). Therefore, changes in Caspase-8 expression, activation status, and functional state may influence the balance among apoptosis-, pyroptosis-, and necroptosis-related signaling pathways.

PANoptosis-like regulatory theory further emphasizes that pyroptosis, apoptosis, and necroptosis can be cross-regulated in inflammatory, infectious, or injury-related environments, suggesting the existence of a composite programmed cell death regulatory network (7, 36–38). In this network, Caspase-8 may serve as an important regulatory node connecting caspase pathways, RIPK/MLKL pathways, and inflammasome-associated signaling. Previous studies using catalytically inactive CASP8 C362S mutant models showed that loss of Caspase-8 catalytic activity can promote necroptosis and pyroptosis, accompanied by ASC speck formation, Caspase-1 activation, and IL-1β secretion, supporting the regulatory role of Caspase-8 in coordinating multiple cell death pathways (11).

In this study, qPCR showed that Casp8 mRNA expression was decreased in the model group but increased in the TF, TO, and TTN groups, while the MP and C8i groups remained at relatively low levels. Western blotting further showed enhanced Caspase-8 and cleaved Caspase-8 expression in the TF, TO, and TTN groups. Together with changes in downstream cleaved Caspase-3, cleaved GSDMD, and p-MLKL, these findings suggest that herbal interventions may modulate cell death-related signaling profiles in GLM lesions through Caspase-8-associated molecular changes.

This study also detected Casp8 C362S-associated amplification signals. The results showed that C362S-associated amplification signals were higher in the model and TF groups and remained relatively high in the MP and C8i groups, whereas the TO group showed the lowest level and the TTN group showed relatively low levels. Combined with increased pyroptosis-related proteins, marked GSDMD membrane accumulation, and pyroptosis-like ultrastructural features in the model group, elevated C362S-associated amplification signals may reflect potential alterations in Caspase-8-associated molecular profiles. Conversely, the TO and TTN groups showed increased Casp8 mRNA and cleaved Caspase-8 expression together with reduced C362S-associated amplification signals, suggesting that these changes may be associated with different Caspase-8-related molecular profiles.

It should be emphasized that qPCR detection of Casp8 mRNA and Casp8 C362S-associated amplification signals cannot directly prove Casp8 C362S mutation status or enzymatic activity. Therefore, the C362S-associated findings should be interpreted as indirect evidence of potential differences in Caspase-8-associated molecular profiles, requiring further confirmation by sequencing, enzymatic activity assays, and functional blockade experiments. Based on the current results, we propose that, under model conditions, decreased Casp8 transcription accompanied by increased C362S-associated amplification signals may be associated with pyroptosis-related inflammatory features in GLM lesions. TO may mainly enhance the Caspase-8/Caspase-3 axis and increase apoptosis-associated molecular features. TTN was associated with increased Casp8 expression, enhanced cleaved Caspase-8 levels, and reduced C362S-associated amplification signals, accompanied by decreased GSDMD-related pyroptosis signals and increased apoptosis- and p-MLKL-associated signals. These molecular changes may contribute to the pathological improvement and tissue remodeling observed after TTN treatment.

### 4.4 TTN regulates local immune cell infiltration rather than simply suppressing inflammation

CD86, CD11c, and CD11b are important markers for evaluating myeloid immune cell activation, recruitment, and functional status. CD86 is mainly expressed on antigen-presenting cells and participates in T-cell activation through CD28 costimulatory signaling. CD11c and CD11b are members of the beta2 integrin family and are commonly expressed in dendritic cells, monocytes/macrophages, and other myeloid cells, where they participate in adhesion, migration, phagocytosis, and local immune regulation (44, 45). Therefore, changes in these markers reflect not only inflammatory cell abundance, but also local myeloid cell recruitment, antigen presentation, and phagocytic clearance functions.

The development of GLM is closely associated with the local mammary immune microenvironment. Recent immunopathological and single-cell studies have shown complex immune cell infiltration and cellular interactions in GLM lesions, with macrophages, T cells, NK cells, epithelial cells, and endothelial cells potentially participating in the maintenance of lesion inflammation and tissue remodeling (24, 25, 46–49). Therefore, GLM treatment does not necessarily equate to simply reducing inflammatory cell numbers, but may depend more on the functional status, recruitment, phagocytic clearance ability, and phenotypic transitions of local immune cells.

Flow cytometry in this study showed significant remodeling of CD86-positive, CD11c-positive, and CD11b-positive myeloid immune cells in mammary tissues of GLM model rats, indicating a persistently activated local immune microenvironment. CD86-positive cells were highest in the TO group, followed by the TF and TTN groups, suggesting that herbal interventions may enhance local myeloid cell activation and antigen presentation-related status (50, 51). CD11c-positive cells were highest in the TO group, followed by the TTN and TF groups, suggesting possible involvement in antigen processing, myeloid cell activation, and inflammatory/clearance-associated phenotypic transition. CD11b-positive cells were highest in the TO group, followed by the TF and TTN groups, suggesting that herbal interventions, especially TO, may enhance monocyte/macrophage recruitment to lesion areas and phagocytic clearance capacity (50, 52).

Notably, CD86-, CD11c-, and CD11b-positive cells were markedly decreased in the MP group, suggesting that glucocorticoids broadly modulate immune cell function and suppress NF-κB-related inflammatory signaling, thereby reducing myeloid cell activation and recruitment (53, 54). However, such nonselective immunosuppression may weaken the clearance of necrotic tissue, antigenic contents, and inflammatory dying cells, thereby impairing effective resolution of chronic inflammatory lesions. Combined with pathological findings, the TTN group did not show simple immune cell suppression, but rather a relatively balanced immune reprogramming profile, which may be more favorable for lesion clearance and tissue repair.

Because flow cytometry is based on single-cell suspensions prepared after tissue digestion, it cannot provide spatial information regarding periductal or interstitial localization. Therefore, this study cannot directly determine the specific tissue origins of CD11c-positive or CD11b-positive cells; these cells should be interpreted as mammary macrophage/myeloid cell-related subsets and phenotypic changes. Future studies combining spatial transcriptomics, multiplex immunofluorescence colocalization, and spatial quantitative analysis are required to clarify the distribution and functional heterogeneity of different myeloid subsets in GLM lesions.

In addition, ELISA showed no significant differences in serum IL-18 and IL-1β levels among groups or time points, suggesting that GLM-related inflammatory changes in this study were mainly concentrated in local mammary lesions rather than reflected by marked systemic cytokine fluctuations. Recent clinical studies indicate that patients with GLM may show abnormalities in immune markers and inflammatory cytokines (55); however, peripheral blood markers do not necessarily fully reflect the local lesion microenvironment. Therefore, local histopathology, immunofluorescence, Western blotting, and flow cytometry may more accurately reflect changes in the GLM lesion microenvironment in this study.

### 4.5 Clinical significance, limitations, and future perspectives

This study explains the potential mechanism of TTN intervention in GLM from the perspective of cell death-related signaling remodeling, providing a concept distinct from simple anti-inflammatory or immunosuppressive therapy. In clinical GLM management, glucocorticoids, immunosuppressants, antibiotics, incision and drainage, surgery, and traditional Chinese medicine are all used, yet prolonged disease course, recurrent abscesses, recurrence, new lesion occurrence, and impaired breast appearance remain clinical challenges (3–5, 12, 13, 26). Conventional strategies often emphasize suppression of inflammation, whereas the present findings suggest that GLM lesion resolution may depends not only on inflammatory intensity, but also on how local inflammatory cells undergo death-related changes and how subsequent tissue remodeling proceeds.

This study has several limitations. First, the findings are based on a rat GLM model, and the regulatory effects of TTN on the Caspase-8-associated PANoptosis-like cell death network require further validation in clinical GLM samples. Second, this study mainly used qPCR, Western blotting, and Caspase-8 inhibitor intervention to observe Caspase-8-related changes, but did not use Casp8 conditional knockout or gene-editing models to verify causality. Given that whole-body Casp8 knockout can cause embryonic lethality in mice (56), tissue- or cell-specific Casp8 conditional knockout models may be more appropriate for future studies. Third, Casp8 C362S-associated amplification signals cannot directly prove Casp8 mutation status or enzymatic activity and require confirmation by sequencing and enzymatic assays. In addition, the flow cytometry and immunofluorescence analyses lacked rigorous spatial localization and colocalization validation. Future studies should combine clinical samples, conditional knockout models, spatial transcriptomics, multiplex immunofluorescence colocalization, and pathway blockade experiments to further clarify the mechanisms underlying TTN-associated regulation of local immune-inflammatory responses and tissue remodeling in GLM. In addition, this study did not systematically evaluate the complete profile of local inflammatory cytokines within mammary lesions. Because the primary focus was Caspase-8-associated PANoptosis-like regulated cell death remodeling, serum IL-1β and IL-18 were included as supportive inflammatory indicators. Future studies using clinical GLM samples and local lesion-level inflammatory mediator analysis are required.

## Data Availability

The original contributions presented in the study are included in the article/**Supplementary Material**. Further inquiries can be directed to the corresponding author.
