# Supplementary material for: Tuoli Tounong decoction promotes lesion resolution in granulomatous lobular mastitis by remodeling a Caspase-8-associated PANoptosis-like cell death network
Source: Front Immunol. 2026 Aug 28;17:1930689. doi: 10.3389/fimmu.2026.1930689 (PMC13561840; doi:10.3389/fimmu.2026.1930689)
Supplement: Supplementary file 1 [file DataSheet1.pdf]

Supplementary Table S1. Quality control information of herbal materials used for Tuolitounong Decoction

Quality control information was obtained from the inspection reports provided for the herbal materials used in Tuolitounong Decoction. All herbal materials were subjected to identification and quality tests according to the Chinese Pharmacopoeia requirements indicated in the corresponding inspection reports.

Herbal material	Dosage in prescription (g)	Manufacturer / supplier shown in report	Batch No.	Origin	Quality standard	Representative quality control results
Ginseng Radix et Rhizoma (人参)	10	Beijing Shengshilong Pharmaceutical Co., Ltd.	260306004	Jilin Baishan	Chinese Pharmacopoeia 2025 edition	Identification consistent; moisture 11.2%; total ash 3.1%; ginsenoside Rg1/Re/Rb1 determined; total ginsenosides 0.43%; compliant
Atractylodis Macrocephalae Rhizoma（白术）	10	Beijing Shengshilong Pharmaceutical Co., Ltd.	260421006	Zhejiang Jinhua	Chinese Pharmacopoeia 2025 edition	Identification consistent; impurities 0.1%; moisture 13.3%; total ash 3.4%; extractive content 47.2%; compliant
Vaccariae Semen (王不留行)	15	Beijing Renwei Pharmaceutical Co., Ltd.	25083103	Hebei Xingtai	Chinese Pharmacopoeia 2020 edition (Parts I and IV)	Identification consistent; impurities 0.4%; moisture 8.6%; hyperoside content 0.23%; pesticide residue compliant
Angelicae Dahuricae Radix (白芷)	10	Beijing Shengshilong Pharmaceutical Co., Ltd.	2512045	Sichuan Suining	Chinese Pharmacopoeia 2025 edition	Identification consistent; impurities 0.3%; moisture 9.4%; total ash 3.3%;

						imperatorin content 0.169%; compliant
Cimicifugae Rhizoma (升麻)	10	Beijing Shengshilong Pharmaceutical Co., Ltd.	2511179	Heilongjiang Mudanjiang	Chinese Pharmacopoeia 2025 edition	Identification consistent; impurities 0.1%; moisture 8.6%; total ash 5.8%; compliant
Glycyrrhizae Radix et Rhizoma (炙甘草)	6	Beijing Shengshilong Pharmaceutical Co., Ltd.	260404001	Gansu Dingxi	Chinese Pharmacopoeia 2025 edition	Identification consistent; impurities 0.1%; moisture 5.7%; total ash 3.1%; glycyrrhizic acid 1.8%; compliant
Angelicae Sinensis Radix (当归)	10	Beijing Xidan Pharmaceutical Co., Ltd.	26050701	Gansu Minxian	Chinese Pharmacopoeia 2025 edition (Parts I and IV)	Identification consistent; impurities 0.3%; moisture 9.8%; total ash 4.5%; quality tests compliant
Astragali Radix (黄芪)	15	Beijing Shengshilong Pharmaceutical Co., Ltd.	260418003	Shanxi Xinzhou	Chinese Pharmacopoeia 2025 edition	Identification consistent; impurities 0.1%; moisture 8.9%; total ash 2.6%; astragaloside IV 0.123%; calycosin glucoside 0.071%; compliant
Gleditsiae Spina (皂角刺)	10	Beijing Xidan Pharmaceutical Co., Ltd.	25051301	Guangxi Yulin	Chinese Pharmacopoeia 2020 edition (Parts I and IV)	Identification consistent; impurities 0.8%; moisture 8.0%; sulfur dioxide residue 17 mg/kg; pesticide residue test compliant

Citri Reticulatae Pericarpium Viride Preparatum (醋青皮)	6	Beijing Sanhe Pharmaceutical Co., Ltd.	52160801	Fujian Nan'an	Chinese Pharmacopoeia 2020 edition	Identification consistent; impurities 0.6%; moisture 6.3%; total ash 4.4%; limonin content reported; pesticide residue compliant

Note: Representative indicators were summarized from the available quality inspection reports, including identification, impurity, moisture, content determination, and safety testing items.

Supplementary Figure S1. Quality inspection reports of herbal materials used in Tuolitounong Decoction

Figure S1-1. Quality inspection report of herbal material

北京盛世龙药业有限公司

检验报告书

原料☐ 辅料☐ 成品 ☑

报告书编号：20260318-3

品名	人参片	规格	片
编号\批号	260306004	装量规格	1kg/袋
产地	吉林白山	检验项目	全检
检验日期	2026 年 03 月 08 日	报告日期	2026 年 03 月 18 日
请验部门	生产车间		
检验依据	《中华人民共和国药典》2025 年版		

检验项目	标准规定	检验结果
【性状】	应与人参片性状相符	与人参片性状相符
【鉴别】		
显微鉴别	应具人参片显微特征	具人参片显微特征
薄层色谱	供试品色谱中，在与对照药材色谱和人参皂苷 Rb1 对照品、人参皂苷 Re 对照品、人参皂苷 Rf 对照品及人参皂苷 Rg1 对照品色谱相应位置上，分别显相同颜色的斑点或荧光斑点	相同
【检查】		
药屑杂质	不得过 3%	0.1%
水分	不得过 12.0%	11.2%
总灰分	不得过 5.0%	3.1%
重金属及有害元素	铅不得过 5mg/kg，	未检出
	镉不得过 1mg/kg，	未检出
	砷不得过 2mg/kg，	0.2mg/kg
	汞不得过 0.2mg/kg，	0.04mg/kg
	铜不得过 20mg/kg。	0.4mg/kg
五氯硝基苯	五氯硝基苯不得过 0.1mg/kg	未检出 g
二氧化硫	二氧化硫残留量不得超过 150mg/kg	未检出
【含量测定】	按干燥品计算，含人参皂苷 Rg1（$C_{42}H_{72}O_{14}$）和人参皂苷 Re（$C_{48}H_{82}O_{18}$）的总量不得少于 0.27%，	0.43%
	人参皂苷 Rb1（$C_{54}H_{92}O_{23}$）不得少于 0.18%	0.22%

备注：该批次重金属及有害元素结果引用原料人参 YG003-2602240 检验结果。

结论：本品按《中华人民共和国药典》2025 年版检验，结果符合规定。

检验人：郭春艳　　复核人：贾春华　　负责人：詹磊

北京盛世龙药业有限公司 质量检验专用章

L-ZL-027-1 检验报告书

Figure S1-2. Quality inspection report of herbal material

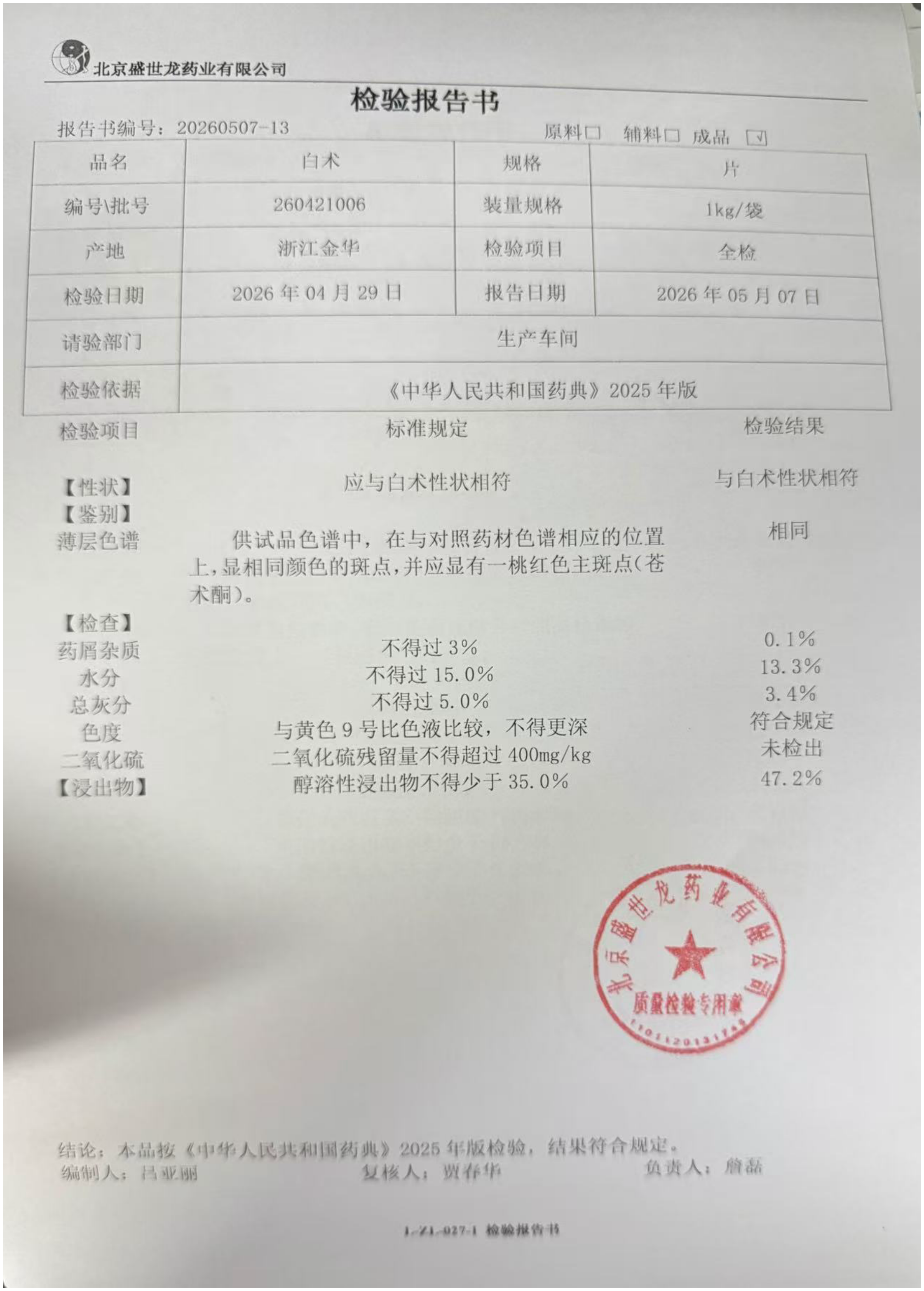

北京盛世龙药业有限公司

检验报告书

报告书编号：20260507-13　　原料☐　辅料☐　成品☑

品名	白术	规格	片
编号\批号	260421006	装量规格	1kg/袋
产地	浙江金华	检验项目	全检
检验日期	2026 年 04 月 29 日	报告日期	2026 年 05 月 07 日
请验部门	生产车间		
检验依据	《中华人民共和国药典》2025 年版		

检验项目	标准规定	检验结果
【性状】	应与白术性状相符	与白术性状相符
【鉴别】薄层色谱	供试品色谱中，在与对照药材色谱相应的位置上，显相同颜色的斑点，并应显有一桃红色主斑点（苍术酮）。	相同
【检查】		
药屑杂质	不得过 3%	0.1%
水分	不得过 15.0%	13.3%
总灰分	不得过 5.0%	3.4%
色度	与黄色 9 号比色液比较，不得更深	符合规定
二氧化硫	二氧化硫残留量不得超过 400mg/kg	未检出
【浸出物】	醇溶性浸出物不得少于 35.0%	47.2%

北京盛世龙药业有限公司 质量检验专用章

结论：本品按《中华人民共和国药典》2025 年版检验，结果符合规定。

编制人：吕亚丽　　复核人：贾春华　　负责人：詹磊

1-Z1-027-1 检验报告书

Figure S1-3. Quality inspection report of herbal material

北京人卫药业有限公司
检验报告单

检验单号：C25090302

品　　名：	炒王不留行	规　　格：	清炒
产　　地：	河北邢台	检品用量：	100g
编号/批号：	25083103	生产日期：	2025-08-31
检品来源：	包装车间	取样日期：	2025-09-01
检验项目：	全检	报告日期：	2025-09-03
检验依据：	《中华人民共和国药典》2020 年版一部 P54 及四部		

检验项目	标准规定	检验结果
性状	应与炒王不留行性状特征相符	与标准相符
薄层鉴别	供试品色谱中，在与王不留行对照药材色谱和王不留行黄酮苷对照品色谱相应的位置上，显相同颜色的荧光斑点。再置碘蒸气中熏至斑点显色清晰，置日光下检视。供试品色谱中，在与王不留行对照药材色谱和刺桐碱对照品色谱相应的位置上，显相同颜色的斑点	相同
药屑杂质	不得过 3%	0.4%
水分	不得过 10.0%	8.6%
浸出物	不得少于 6.0%	7.9%
含量测定	含王不留行黄酮苷（$C_{32}H_{38}O_{19}$）不得少于 0.15%	0.23%
二氧化硫残留量	不得过 150mg/kg	0mg/kg
*33 种禁用农药	不得检出（不得过定量限）	未检出（未过定量限）

*33 种禁用农药 详见报告编号：Y25082603

以下空白

检验结论：本品按《中华人民共和国药典》2020 年版检验上述项目，结果符合规定。

报告人：　　　复核人：　　　批准人：

Figure S1-4. Quality inspection report of herbal material

北京盛世龙药业有限公司

检验报告书

报告书编号：20251215-12　　　　原料☐　辅料☐　成品 ☑

品名	白芷	规格	片
编号\批号	2512045	装量规格	1kg/袋
产地	四川遂宁	检验项目	全检
检验日期	2025 年 12 月 10 日	报告日期	2025 年 12 月 15 日
请验部门	生产车间		
检验依据	《中华人民共和国药典》2025 年版		

检验项目	标准规定	检验结果
【性状】	应与白芷性状相符	与白芷性状相符
【鉴别】		
显微鉴别	应具白芷显微特征	具白芷显微特征
薄层色谱	供试品色谱中，在与对照药材色谱和欧前胡素对照品、异欧前胡素对照品色谱相应的位置上，显相同颜色的荧光斑点。	相同
【检查】		
药屑杂质	不得过 3%	0.3%
水分	不得过 14.0%	9.4%
总灰分	不得过 5.0%	3.3%
二氧化硫	二氧化硫残留量不得超过 150mg/kg	未检出
【浸出物】	醇溶性浸出物不得少于 15.0%	20.5%
【含量测定】	按干燥品计算，含欧前胡素（$C_{16}H_{14}O_4$）不得少于 0.080%。	0.169%

结论：本品按《中华人民共和国药典》2025 年版检验，结果符合规定。

检验人：郭春艳　　　　复核人：贾春华　　　　负责人：詹磊

L-ZL-027-1 检验报告书

Figure S1-5. Quality inspection report of herbal material

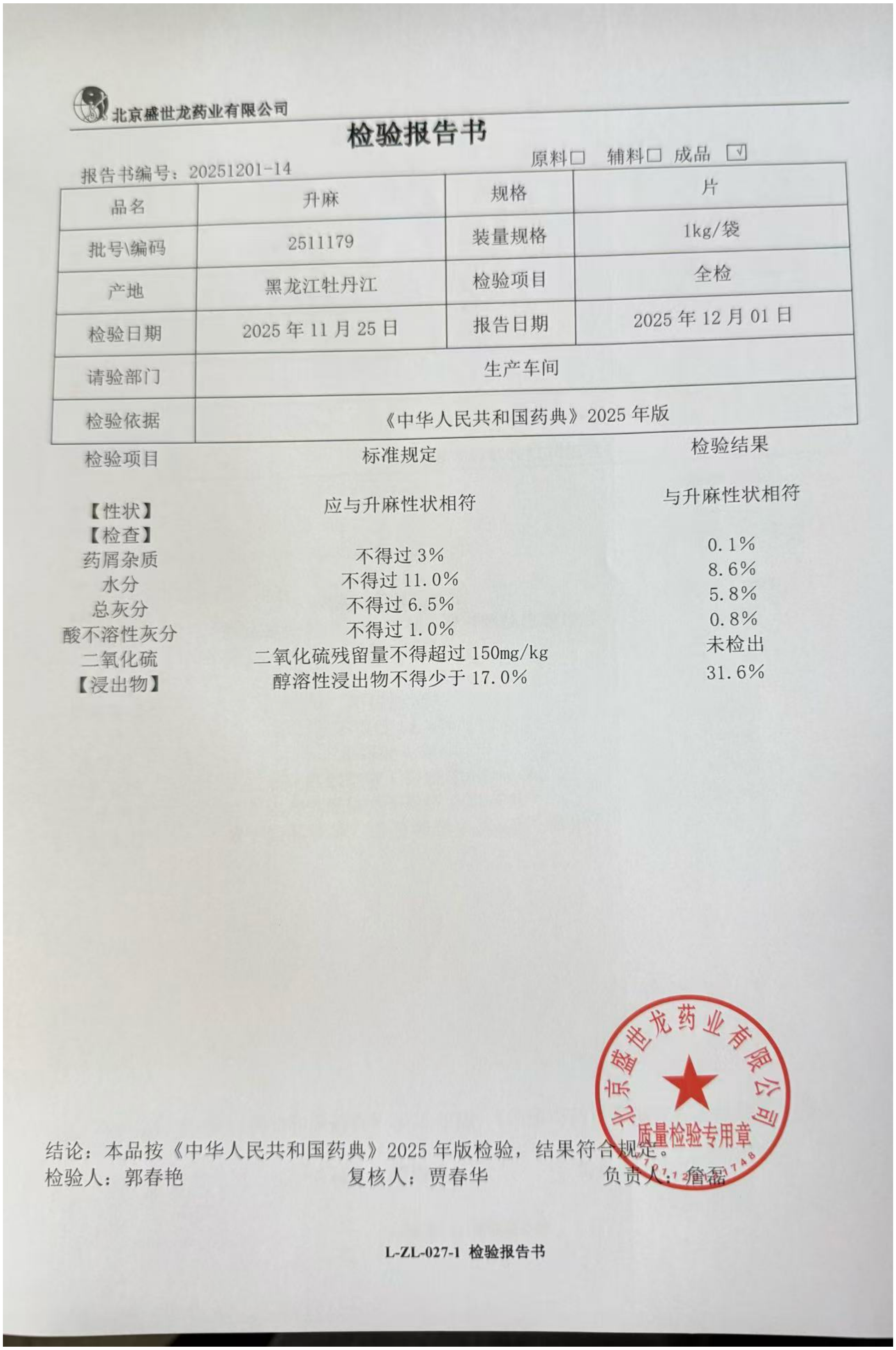

北京盛世龙药业有限公司

检验报告书

报告书编号：20251201-14　　原料☐　辅料☐　成品 ☑

品名	升麻	规格	片
批号\编码	2511179	装量规格	1kg/袋
产地	黑龙江牡丹江	检验项目	全检
检验日期	2025 年 11 月 25 日	报告日期	2025 年 12 月 01 日
请验部门	生产车间		
检验依据	《中华人民共和国药典》2025 年版		

检验项目	标准规定	检验结果
【性状】	应与升麻性状相符	与升麻性状相符
【检查】		
药屑杂质	不得过 3%	0.1%
水分	不得过 11.0%	8.6%
总灰分	不得过 6.5%	5.8%
酸不溶性灰分	不得过 1.0%	0.8%
二氧化硫	二氧化硫残留量不得超过 150mg/kg	未检出
【浸出物】	醇溶性浸出物不得少于 17.0%	31.6%

结论：本品按《中华人民共和国药典》2025 年版检验，结果符合规定。

检验人：郭春艳　　复核人：贾春华　　负责人：詹磊

北京盛世龙药业有限公司 质量检验专用章

L-ZL-027-1 检验报告书

Figure S1-6. Quality inspection report of herbal material

北京盛世龙药业有限公司

检验报告书

报告书编号：20260423-1　　原料□　辅料□　成品 ☑

品名	炙甘草	规格	蜜炙
编号\批号	260404001	装量规格	1kg/袋
产地	甘肃定西	检验项目	全检
检验日期	2026 年 04 月 18 日	报告日期	2026 年 04 月 23 日
请验部门	生产车间		
检验依据	《中华人民共和国药典》2025 年版		

检验项目	标准规定	检验结果
【性状】	应与炙甘草性状相符	与炙甘草性状相符
【鉴别】		
薄层色谱	供试品色谱中，在与对照药材色谱相应的位置上，显相同颜色的荧光斑点；在与甘草酸铵盐对照品色谱相应的位置上，显相同的橙黄色荧光斑点。	相同
【检查】		
药屑杂质	不得过 3%	0.1%
水分	不得过 10.0%	5.7%
总灰分	不得过 5.0%	3.1%
二氧化硫	二氧化硫残留量不得超过 150mg/kg	未检出
【含量测定】	按干燥品计算，含甘草苷($C_{21}H_{22}O_9$)不得少于 0.45%，甘草酸($C_{42}H_{62}O_{16}$)不得少于 1.0%。	0.60%<br>1.8%

结论：本品按《中华人民共和国药典》2025 年版检验，结果符合规定。

编制人：吕亚丽　　复核人：贾春华　　负责人：詹磊

Figure S1-7. Quality inspection report of herbal material

北京西单医药有限责任公司

中药饮片检验报告单

检验单号：C26051402

品　　名：	当归	批　　号：	26050701
产　　地：	甘肃省岷县	规　　格：	选、薄片
生产日期：	2026-05-07	样品数量：	100g
检验目的：	出厂检验	检验项目：	全项检验☑　部分检验☐
收样日期：	2026-05-09	报告日期：	2026-05-14

检验依据：《中国药典》2025 年版一部、四部

检验项目	标准规定	检验结果
性状	应与当归性状相符	与当归性状相符
显微鉴别	应与当归显微特征相符	与当归显微特征相符
薄层鉴别	供试品色谱中，在与对照药材色谱相应的位置上，显相同颜色的荧光斑点	符合规定
	供试品色谱中，在与对照品色谱相应的位置上，显相同颜色的荧光斑点	符合规定
杂质药屑	不得过 3%	0.3%
水分	不得过 15.0%	9.8%
总灰分	不得过 7.0%	4.5%
酸不溶性灰分	不得过 2.0%	[illegible]1.3%
*重金属及有害元素（引用药材 YL26041402 检验数据）	铅不得过 5mg/kg；镉不得过 1mg/kg；砷不得过 2mg/kg；汞不得过 0.2mg/kg；铜不得过 20mg/kg。	铅 [illegible]mg/kg；镉 [illegible]mg/kg；砷 0mg/kg；汞 0.0mg/kg；铜 [illegible]5mg/kg。
浸出物	不得少于 45.0%	[illegible]
二氧化硫残留量	不得过 150mg/kg	[illegible]7.3mg/kg
*47 种禁用农药残留（引用药材 YL26041402 检验数据）	应符合规定	符合规定

以下空白

*表示委托安徽微谱检测技术有限公司检测，报告编号为 BZA50-26044122-JC-02。

检验结论：本品按《中国药典》2025 年版一部、四部检验依据检验，结果符合规定。

签发人：[illegible]

Figure S1-8. Quality inspection report of herbal material

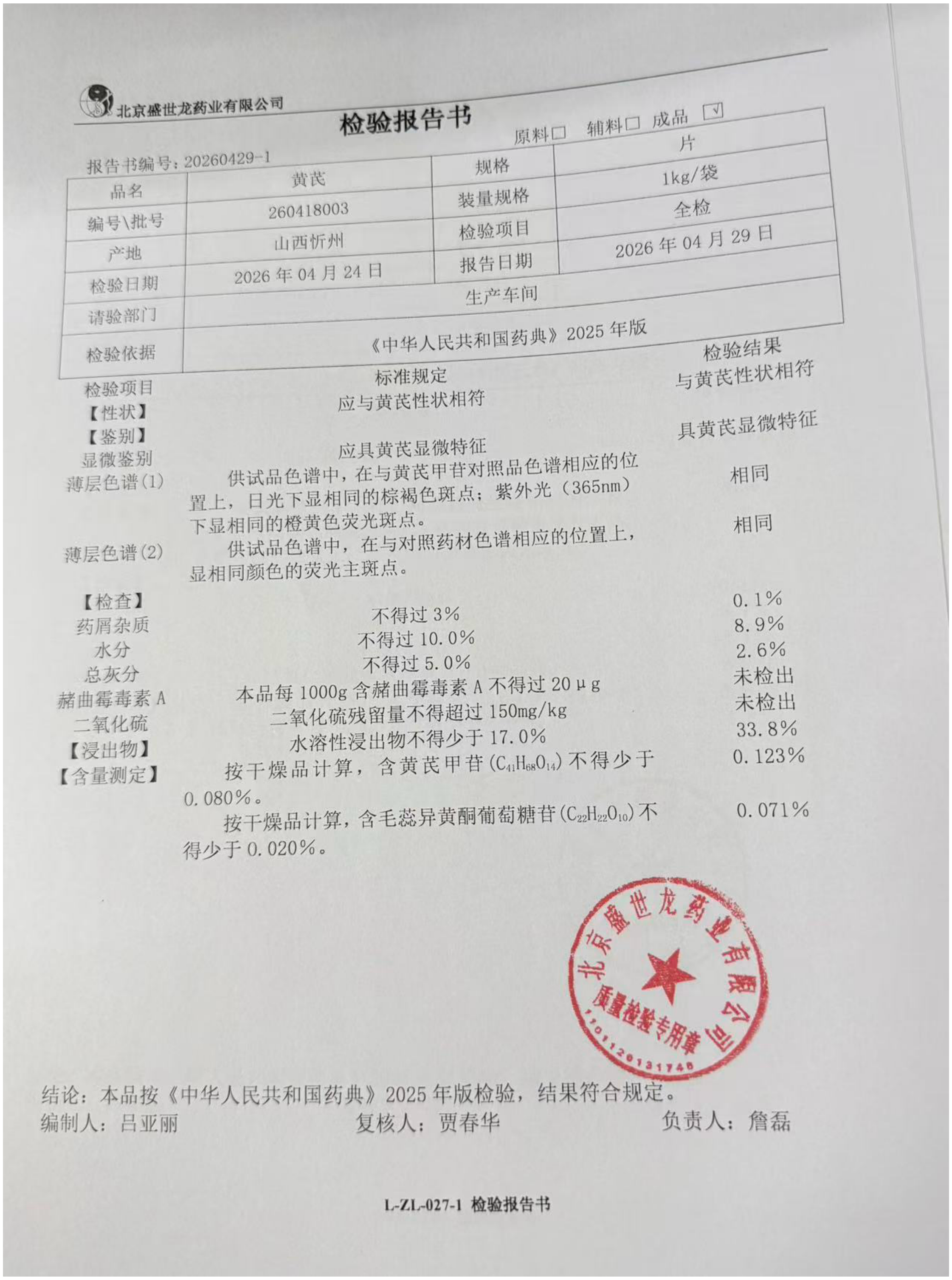

北京盛世龙药业有限公司

检验报告书

原料☐ 辅料☐ 成品 ☑

报告书编号：20260429-1

品名	黄芪	规格	片
编号\批号	260418003	装量规格	1kg/袋
产地	山西忻州	检验项目	全检
检验日期	2026 年 04 月 24 日	报告日期	2026 年 04 月 29 日
请验部门	生产车间		
检验依据	《中华人民共和国药典》2025 年版		

检验项目	标准规定	检验结果
【性状】	应与黄芪性状相符	与黄芪性状相符
【鉴别】		
显微鉴别	应具黄芪显微特征	具黄芪显微特征
薄层色谱(1)	供试品色谱中，在与黄芪甲苷对照品色谱相应的位置上，日光下显相同的棕褐色斑点；紫外光（365nm）下显相同的橙黄色荧光斑点。	相同
薄层色谱(2)	供试品色谱中，在与对照药材色谱相应的位置上，显相同颜色的荧光主斑点。	相同
【检查】		
药屑杂质	不得过 3%	0.1%
水分	不得过 10.0%	8.9%
总灰分	不得过 5.0%	2.6%
赭曲霉毒素 A	本品每 1000g 含赭曲霉毒素 A 不得过 20μg	未检出
二氧化硫	二氧化硫残留量不得超过 150mg/kg	未检出
【浸出物】	水溶性浸出物不得少于 17.0%	33.8%
【含量测定】	按干燥品计算，含黄芪甲苷($C_{41}H_{68}O_{14}$)不得少于 0.080%。	0.123%
	按干燥品计算，含毛蕊异黄酮葡萄糖苷($C_{22}H_{22}O_{10}$)不得少于 0.020%。	0.071%

北京盛世龙药业有限公司 质量检验专用章

结论：本品按《中华人民共和国药典》2025 年版检验，结果符合规定。

编制人：吕亚丽　　复核人：贾春华　　负责人：詹磊

L-ZL-027-1 检验报告书

Figure S1-9. Quality inspection report of herbal material

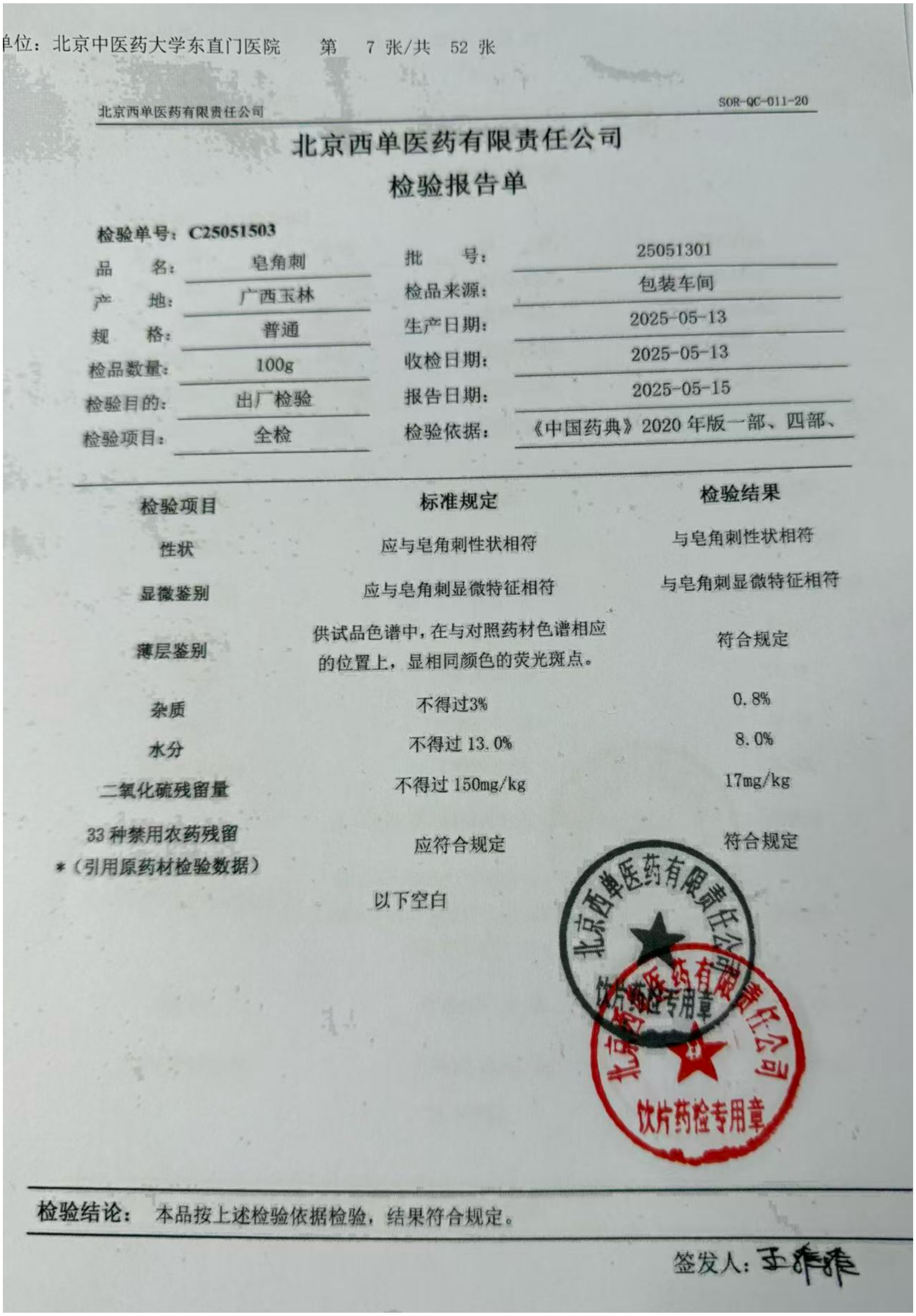

单位：北京中医药大学东直门医院　　第　7 张/共　52 张

北京西单医药有限责任公司　　SOR-QC-011-20

北京西单医药有限责任公司
检验报告单

检验单号：C25051503

品　　名：	皂角刺	批　　号：	25051301
产　　地：	广西玉林	检品来源：	包装车间
规　　格：	普通	生产日期：	2025-05-13
检品数量：	100g	收检日期：	2025-05-13
检验目的：	出厂检验	报告日期：	2025-05-15
检验项目：	全检	检验依据：	《中国药典》2020 年版一部、四部、

检验项目	标准规定	检验结果
性状	应与皂角刺性状相符	与皂角刺性状相符
显微鉴别	应与皂角刺显微特征相符	与皂角刺显微特征相符
薄层鉴别	供试品色谱中，在与对照药材色谱相应的位置上，显相同颜色的荧光斑点。	符合规定
杂质	不得过3%	0.8%
水分	不得过 13.0%	8.0%
二氧化硫残留量	不得过 150mg/kg	17mg/kg
33 种禁用农药残留 *（引用原药材检验数据）	应符合规定	符合规定
	以下空白	

北京西单医药有限责任公司 饮片药检专用章

北京西单医药有限责任公司 饮片药检专用章

检验结论：　本品按上述检验依据检验，结果符合规定。

签发人：王乖乖

Figure S1-10. Quality inspection report of herbal material

北京三和药业有限公司　　　　编号:SOR/QC/005-03

成品检验报告书

报告书编号：CP202508043

品　　名	醋青皮	产　　地	福建省南安市
规　　格	醋炙	装　　量	1kg/袋
批　　号	52160801	检验日期	2025 年 08 月 16 日
检品数量	150g	报告日期	2025 年 08 月 21 日
检验依据	《中国药典》2020 年版		

检验项目	标准规定	检验结果
【性状】	应与醋青皮性状相符	与醋青皮性状相符
【鉴别】		
显微鉴别	应具青皮显微特征	具青皮显微特征
薄层鉴别	在供试品色谱中，与橙皮苷对照品色谱相应的位置上，显相同颜色的荧光斑点。	相同
【检查】		
杂质	不得过 3.0%	0.6%
水分	不得过 11.0%	6.3%
总灰分	不得过 6.0%	4.4%
二氧化硫残留量	不得过 150mg/kg	39mg/kg
【含量测定】		
本品含橙皮苷（$C_{28}H_{34}O_{15}$）	不得少于 3.0%	18.5%
33 种禁用农药残留量	不得检出（不得过定量限）	符合规定

结论：本品按《中国药典》2020 年版检验，结果符合规定。

检验人：于倩　　复核人：岳琳双　　负责人：贾王俊

（印章：北京三和药业有限公司 检验专用章）
